# Expanding the Range of Bioorthogonal Tags for Multiplex Stimulated Raman Scattering Microscopy

**DOI:** 10.1002/anie.202311530

**Published:** 2023-10-27

**Authors:** Neville Murphy, William J. Tipping, Henry J. Braddick, Liam T. Wilson, Nicholas C. O. Tomkinson, Karen Faulds, Duncan Graham, Pau Farràs

**Affiliations:** ^1^ School of Biological and Chemical Sciences University of Galway Galway H91CF50 Ireland; ^2^ CÚRAM, The SFI Research Centre for Medical Devices University of Galway Galway H91 W2TY Ireland; ^3^ Centre for Molecular Nanometrology WestCHEM Department of Pure and Applied Chemistry, Technology and Innovation Centre University of Strathclyde Glasgow G1 1RD United Kingdom; ^4^ Department of Pure and Applied Chemistry University of Strathclyde Glasgow G1 1XL United Kingdom

**Keywords:** Bioorthogonal Labeling, Imaging Probes, Raman Microscopy, Spectral Phasor Analysis, Stimulated Raman Scattering

## Abstract

Multiplex optical detection in live cells is challenging due to overlapping signals and poor signal‐to‐noise associated with some chemical reporters. To address this, the application of spectral phasor analysis to stimulated Raman scattering (SRS) microscopy for unmixing three bioorthogonal Raman probes within cells is reported. Triplex detection of a metallacarborane using the B−H stretch at 2480–2650 cm^−1^, together with a *bis*‐alkyne and deuterated fatty acid can be achieved within the cell‐silent region of the Raman spectrum. When coupled to imaging in the high‐wavenumber region of the cellular Raman spectrum, nine discrete regions of interest can be spectrally unmixed from the hyperspectral SRS dataset, demonstrating a new capability in the toolkit of multiplexed Raman imaging of live cells.

## Graphical abstract

Hyperspectral stimulated Raman scattering (SRS) microscopy with spectral phasor analysis enables the multiplex detection of bioorthogonal Raman groups in cells. Unmixing of the overlapping alkyne and deuterium signals together with the detection of metallacarboranes is reported within the cell‐silent region of the Raman spectrum.

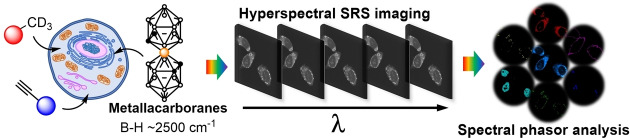

Multispectral imaging is emerging as a powerful tool for the simultaneous visualisation of multiple intracellular targets, which is critical for understanding the interconnected nature of complex biological systems. For example, multispectral fluorescence imaging enables the detection of several intracellular labels, whilst the application of spectral phasor analysis enables the unmixing of up to seven fluorescent signals within the same sample.^[1]^ The narrow spectral frequencies of IR and Raman bands (≈15 cm^−1^) compared to fluorescence emission (≈1500 cm^−1^) greatly facilitates highly multiplexed detection.^[2]^ When coupled to stimulated Raman scattering (SRS) microscopy, the fast image acquisition rates and high spatial resolution required for cellular imaging studies are possible.^[3]^ To extend the multiplex capability of hyperspectral SRS imaging, data analysis techniques have enabled the unmixing of overlapping spectral features in the C−H stretching region of the Raman spectrum (2800–3100 cm^−1^).^[4]^ A promising approach is the use of spectral phasor analysis in hyperspectral SRS imaging datasets which has become a high‐content platform for cellular visualisation,^[5]^ SRS‐based cytometry^[6]^ and for investigating drug‐cell interactions.^[7]^ However, the unmixing of signals derived from Raman labels in a cellular sample remains a challenge, which has the potential to give access to higher order multiplexing without the requirement for label redesign and synthesis.

Current strategies for multiplex SRS detection use bioorthogonal Raman groups^[8]^ which can be tuned to discrete vibrational frequencies by chemical functionalisation, isotopic editing and end‐capping substitution of polyynes.^[9]^ The current palette of Raman labels covers the range 2000–2300 cm^−1^ of the Raman spectrum.^[10]^ We propose that to extend the multiplexing capability of SRS microscopy, spectral phasor analysis could be used to unmix overlapping signals of Raman labels, which has proven to be a fruitful method in fluorescence^[1]^ and bioluminescence imaging.^[11]^ In addition, vibrational probes with frequencies in the uncharted region of the cell‐silent window (2300–2800 cm^−1^) are yet to be fully explored through multiplex imaging using SRS microscopy.^[12]^ The B−H stretching mode of metallacarboranes is detected at 2570 cm^−1^ in the Raman spectrum,^[13]^ which is spectrally isolated from other bioorthogonal Raman groups. We therefore reasoned that with the application of hyperspectral SRS microscopy and spectral phasor analysis, the first example of multiplex detection via spectral unmixing in tandem with metallacarboranes and alternative bioorthogonal Raman groups could be realised. This would provide a significant new entity in the toolkit of Raman tags for multiplex biological analysis.

Herein, we present the first application of metallacarboranes as novel bioorthogonal groups for cellular visualisation using SRS microscopy. Using spectral phasor analysis, we demonstrate multiplex detection of nine regions of interest that supersedes recent fluorescence capability. This study highlights the potential of exploiting vibrational frequencies in the uncharted region of the cell‐silent window to provide simultaneous, multiplex detection in live cell systems.

Boron clusters such as the dicarba‐*closo*‐dodecaboranes and metallacarboranes have been candidates for biomedical applications for many years, owing to properties such as unique biological interactions, high stability and relatively low toxicity typically associated with these compounds.^[14]^ Metallacarboranes are a family of boron clusters that are comprised of boron, hydrogen, carbon and a metal centre.^[15]^ This work contains data from icosahedral metallacarboranes; two dicarbollide cages as ligands to the metal centre, and the *ortho‐*dicarba‐*closo*‐dodecaborane, (Figure 1A) derivatives of which have previously been shown to readily cross biological membranes.^[16]^ To demonstrate the potential of metallacarboranes for multiplex detection with alkynes and deuterated molecules in a single cell using SRS microscopy, we first investigated the impact of varying the identity and oxidation state of the metal centre on the B−H vibrational modes. The cage B−Hs have been shown to have a bearing on their ability to cross cell membranes, while indications that their hydridity is affected by the metal centre can be found in the literature.^[17]^ The delivery and localisation of intratumoural boron concentrations in the therapeutic window of 15–30 μg/g tumour represents a major challenge in boron neutron capture therapy (BNCT), despite significant research effort to do so.^[18]^ Here, we sought to investigate the intracellular localisation of metallacarboranes using high‐resolution SRS imaging to understand this challenge. In doing so, we demonstrate the versatility of metallacarboranes as contrast agents for multiplex SRS microscopy. Raman spectra were acquired from the *ortho*‐carborane (**1**) together with the monoanionic metallacarboranes (**2**) that incorporated different metal centres (M=Co, Cr, Fe, and Ni) in solid form (Figure 1A).^[19]^ The metallacarboranes were prepared using a standard procedure (see Scheme S1–2, ESI). In addition, we investigated the use of two butadiyne probes, AM‐ester (**3**) which contains an acetoxymethyl ester group that is hydrolysed by intracellular esterases,^[20]^ and imidazole (**4**) that is an unsymmetrical butadiyne flanked by a 5‐ and 6‐membered ring system (Figure 1B). AM‐ester **3** has been recently reported as a ratiometric alkyne sensor for cellular esterase enzyme,^[20]^ which was selected to show the multiplex imaging possibilities of metallacarboranes with ratiometric alkyne sensors. There is currently a lack of available Raman probes that localise to the nucleus, as many butadiynes are non‐polar and have been detected within the cytoplasm of cells. We therefore we designed imidazole **4** which is a polar unsymmetrical butadiyne as a novel, nuclear targeting Raman probe for use in this study.

**Figure 1:**
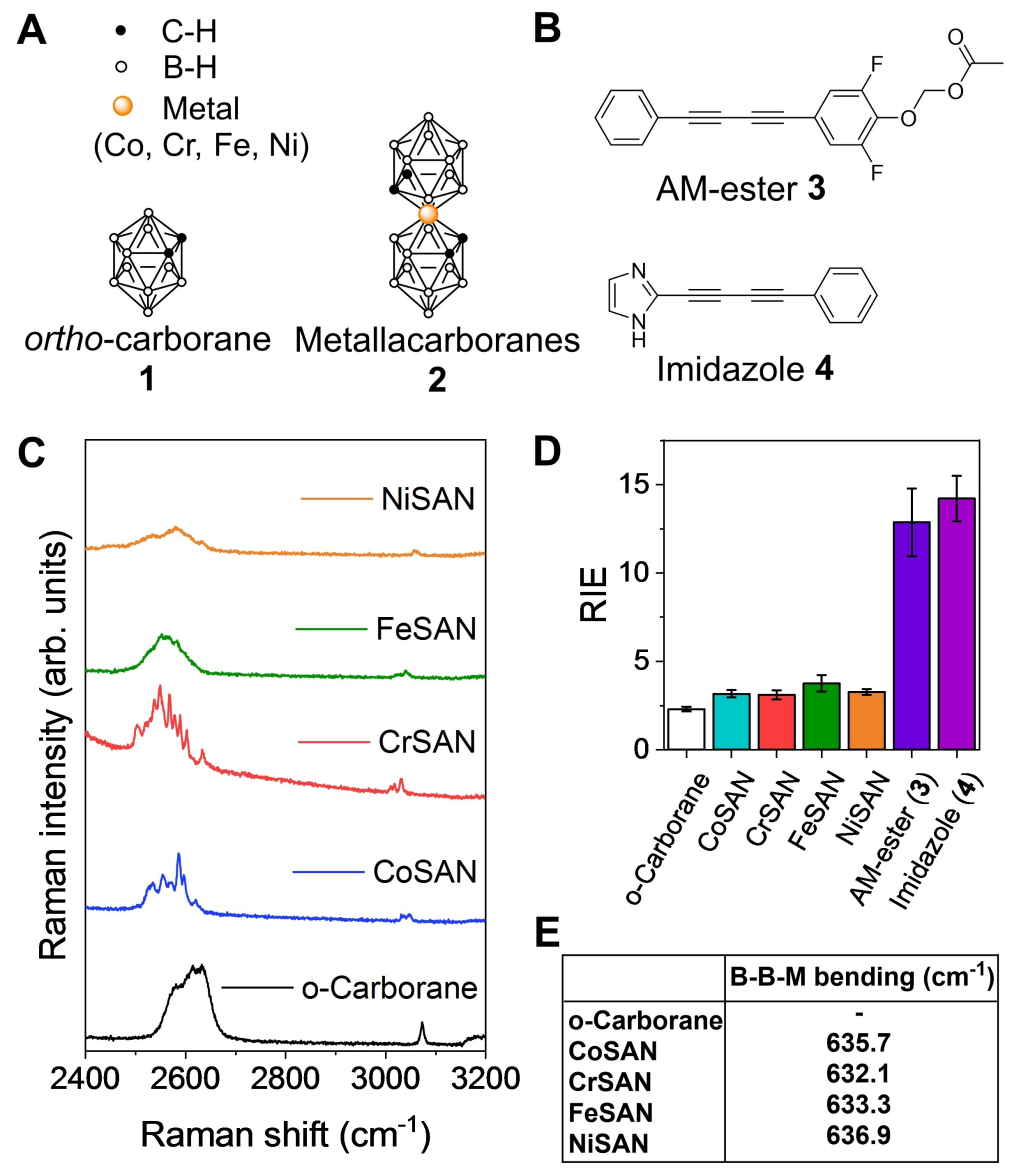
Analysis of metallacarboranes using spontaneous Raman scattering. A) Molecular structure of metallacarboranes used in this study. B) Chemical structures of the *bis*‐alkynes used in this study. C) Raman spectra of ortho‐carborane and the metallacarboranes in solid form. Spectra were acquired using 785 nm excitation with a 20× lens (~20 mW) for 10 s. D) Analysis of the relative Raman scattering of metallacarboranes (B−H, 2480–2630 cm^−1^) and *bis*‐alkynes (C≡C, ~2220 cm^−1^) relative to EdU (5‐ethynyl‐2′‐deoxyuridine, C≡C, 2120 cm^−1^). Data represent the mean relative intensity vs. EdU (RIE) from 12 replicate spectra with error bars ±S.D. E) Raman shift of the B−B−M bending mode of metallacarboranes.

Raman spectra acquired using NIR excitation are presented in Figure 1C, with the corresponding fingerprint regions presented in Figure S1. The B−H stretching vibrations from boron clusters are represented by a broad peak at 2480–2680 cm^−1^ in the Raman spectrum. The broad B−H stretching modes of the metallacarboranes show some peak splitting that is particularly evident for CoSAN and CrSAN. The potential reasons for B−H peak splitting include the chemical inequivalence of the B−H environments across the metallacarborane, including the impact of natural isotopic variation of boron (^11^B and ^10^B).^[21]^ In addition, partial resolution of the B−H bands belonging to different symmetry species and the prevalence of crystal effects have also been reported.^[21]^

As a standard approach for bioorthogonal Raman probes,^[22]^ we determined the intensity of ν(B−H) stretching relative to 5‐ethynyl‐2′‐deoxyuridine, EdU, to enable direct comparison with other Raman probes (Figure 1D). The metallacarboranes have relative intensity to EdU (RIE) values of 3, which is greater than the *ortho*‐carborane, and is, in part due to the sandwich complex formed from the coordination of a metal centre with two dicarbollide clusters. When compared to the RIE values of the butadiynes **3** and **4**, metallacarboranes generated approximately 4‐fold lower RIE values.

Based on a previous computational study of CoSAN,^[23]^ most of the Raman bands likely originate from the carboranyl component. The coordination of different metal centres results in shifting of several key vibrational modes within the Raman spectrum. For example, a whole molecule stretching mode is detected at ≈200 cm^−1^ for the metallacarboranes which was noticeably absent in the Raman spectrum of *ortho*‐carborane (Figure S1 and Table S1), while the B−B−M bending mode (≈630–640 cm^−1^, Figure 1E) and the dicarbollide cage rocking mode (220–280 cm^−1^) were shown to be sensitive to different metal centres. The partial deuteration of CoSAN to generate CoSAN‐D_2_ (Scheme S3) resulted in C−D stretching modes detected at 2260.4 and 2273.4 cm^−1^ (Figure S2), representing approximately a 750 cm^−1^ red shift when compared to the C−H stretching modes (Table S2). The application of CoSAN‐D_2_ demonstrated the potential for multiplex imaging using C−D and B−H vibrations.

Having established the vibrational spectral features of metallacarboranes, we then studied their intracellular distribution using SRS microscopy. The sodium salt of the metallacarboranes were readily soluble in aqueous‐based culture medium, which is an attractive feature for Raman labelling studies. Live HeLa cells were treated with metallacarboranes (250 μM, 4 h) before SRS images were acquired at 2930 cm^−1^ (CH_3_ symmetric stretch) and 2851 cm^−1^ (CH_2_ symmetric stretch), respectively, which represent label‐free markers of the cellular protein and lipid pools (Figure 2A).^[24]^ Imaging at 2570 cm^−1^, representing the mid‐point of B−H stretching modes, enabled the intracellular visualisation of the metallacarboranes which were found to be predominantly localised in the cell cytoplasm.

**Figure 2:**
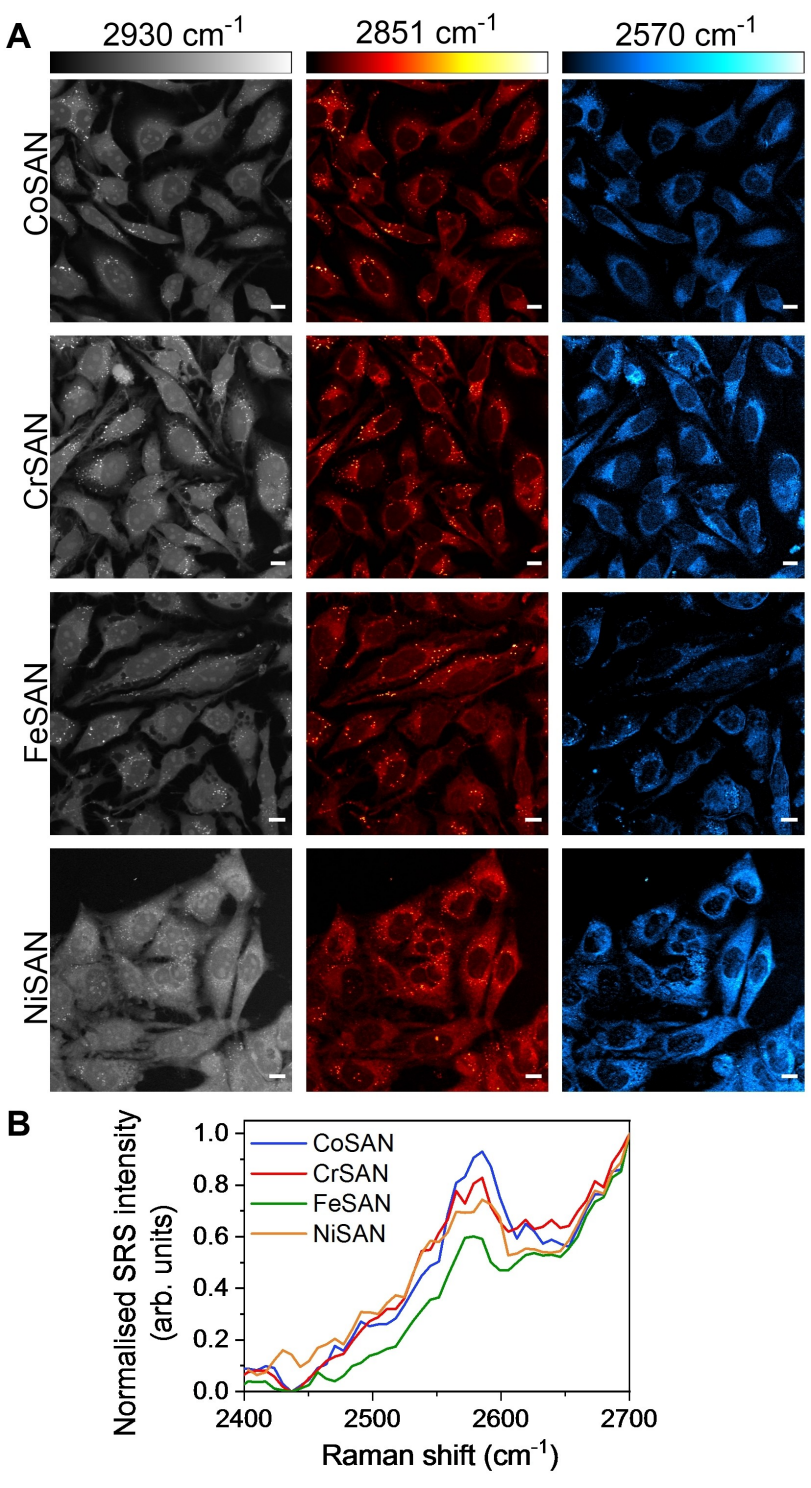
Imaging metallacarboranes in live cells using SRS microscopy. A) HeLa cells were treated with metallacarborane (250 μM, 4 h) before imaging using SRS microscopy at the following frequencies: 2930 cm^−1^ (CH_3_, proteins), 2851 cm^−1^ (CH_2_, lipids), 2570 cm^−1^ (B−H) with 2400 cm^−1^ (off‐resonance) signal subtracted. DMSO control cells: see Figure S3. Scale bars: 10 μm. B) SRS spectra are also presented for single cells across the range 2400–2700 cm^−1^. Spectra normalised between 0–1.

Wavelength scanning SRS images were acquired across the range 2700–2400 cm^−1^ by re‐tuning the pump laser wavelength by 0.4 nm (≈7 cm^−1^) between each image frame. The resulting SRS spectra indicated a significant B−H stretching mode was detected for all metal centres (Figure 2B). As such, these images represent the first detection of metallacarboranes using SRS microscopy, with a reduced treatment concentration compared to previous Raman‐based studies which used 2–25 mM treatment concentrations.^[13]^ We acquired SRS images of HeLa cells treated with varying concentrations of CoSAN (250 μM‐1 mM) at different timepoints (Figure S3). HeLa cells treated with CoSAN (1 mM, 15 min) showed significant accumulation of the metallacarborane throughout the cell, and in particular, accumulation was detected in the cell nucleoli (Figure S3A−C). This pattern of accumulation is surprising given that the biomolecular content of these two regions of the cell are markedly different. However, at this higher concentration, cellular cytotoxicity was observed: the cells showed membrane blebbing and rupture as indicated by the yellow arrowheads, which was likely due to the increased accumulation of the CoSAN (Figure S3C) and a reduction in cell viability (Figure S3D). Altogether, these results indicated that the intracellular localisation of metallacarboranes in HeLa cells is concentration dependant, such that, at <1 mM extracellular concentrations, a predominantly cytoplasmic distribution was observed, and cell viability was largely unaffected at 250–400 μM for 4 h. In these experiments, an advantage of a Raman imaging approach is that the direct detection of the B−H stretch is achieved without the use of fluorophore labelling^[25]^ or nanoparticle enhancement.^[26]^ At treatment concentrations >1 mM, the localisation of the CoSAN appears to be pan‐cellular which agrees with the observations made using Raman imaging albeit in a different cell line (HEK293 cells).^[13]^ In our study, the use of SRS microscopy, that offers greater sensitivity detection compared to Raman scattering, enabled the detection of CoSAN at lower extracellular concentrations which resulted in a lower cytotoxic burden (Figure S3).

Hyperspectral SRS imaging enables biomolecular characterization based directly on the SRS spectrum.[^5^, ^6^, ^7^] Spectral phasor analysis is a Fourier transform‐based method to enable cellular segmentation directly from the SRS spectrum at each pixel within the image stack.[^5^, ^6^, ^7^] We applied spectral phasor analysis for the spectral unmixing of different bioorthogonal Raman bands within the cell‐silent region of the spectrum to facilitate multiplex detection. To do so, we first investigated the single‐plex detection of FeSAN in HeLa cells using spectral phasor analysis. HeLa cells were treated with FeSAN (500 μM, 15 min) before SRS images were acquired across the range 3050–2800 cm^−1^ (C−H vibrations) and 2650–2450 cm^−1^ (B−H vibrations). Spectral phasor analysis was first performed on the region 3050–2800 cm^−1^ (Figure 3A). The spectral phasor plot was segmented into regions of interest (ROIs) for cellular characterisation which were ascribed as follows: (A) nucleus, (B) nucleolus, (C) cytoplasm, (D) lipid droplets (LDs), (E) LD periphery and (F) cell boundary. Thus, spectral phasor analysis allows for label‐free cellular visualisation, and identification of discrete intracellular regions without the use of organelle trackers and molecular staining protocols. The spectral phasor analysis of the B−H stretching regions indicated a diffuse region, which identified the distribution of FeSAN in the cytoplasm and nucleolus (Figure 3B). The accumulation of FeSAN is likely a reflection of the amphipathic nature of the molecule.^[27]^ Moreover, a similar distribution pattern was observed in the single‐plex spectral phasor analysis of NiSAN, which has previously garnered attention due to its redox and molecular motor properties (Figure S4).^[28]^

**Figure 3:**
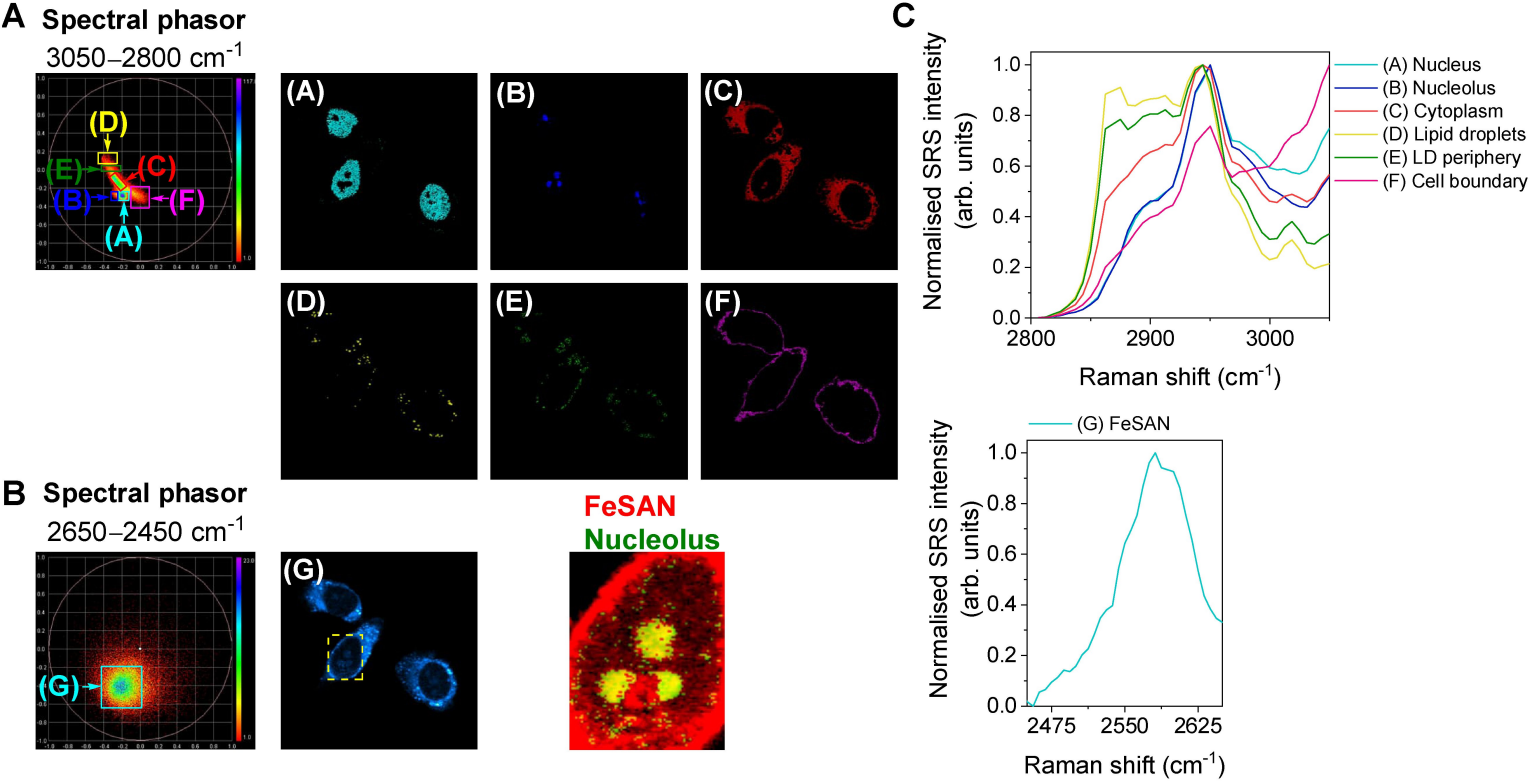
Cellular segmentation using spectral phasor analysis. HeLa cells were treated with FeSAN (500 μM, 15 min) before SRS images were acquired across the range A) 3050–2800 cm^−1^ (0.4 nm, 40 images) and B) 2650–2450 cm^−1^ (0.4 nm, 35 images). The spectral phasor plot has been segmented into the following regions: (A) nucleus, (B) nucleolus, (C) cytoplasm, (D) lipid droplets (LDs), (E) LD periphery, (F) cell boundary and (G) FeSAN. A yellow dashed marker indicates the expanded region showing co‐localization of the (G) FeSAN segment (red) with the (B) nucleolus segment (green). C) Normalised SRS spectra corresponding to the segments (A)–(G). The SRS spectra have been normalised between 0–1.

We next applied spectral phasor analysis to study a triplex of bioorthogonal Raman groups in the same cellular population, this time deploying CrSAN, which has previously been investigated as an electrochemical sensing agent as the metallacarborane component.^[29]^ To do so, HeLa cells were treated with stearic acid‐*d_35_* **5** (200 μM, 8 h) before washing and secondary treatment with CrSAN (250 μM, 4 h) and AM‐ester **3** (100 μM, 30 min). Given the differences in the Raman scattering cross section and relative intensities compared to EdU (RIE) of C−D, C≡C and B−H bonds, we found it necessary to use different treatment concentrations (and times) in order to achieve a detectable intracellular signal for each probe. SRS spectra were acquired across the range 3050–2800 cm^−1^ for cellular segmentation, together with 2650–2446.3 cm^−1^ (B−H) and 2250–2000 cm^−1^ (C≡C and C−D). The resultant spectral phasor plot is presented in Figure 4 which was then segmented as before (A−F). CrSAN was detected throughout the cell cytoplasm and weakly associated with the nucleus (G). Furthermore, two discrete regions of the spectral phasor plot identified (H) as a mixture of stearic acid‐*d_35_* **5** (CD_2_ stretch, 2102 cm^−1^ see Figure S5) and AM‐ester **3** (2220 cm^−1^ see Figure S5) which were co‐localised, and (I) which was predominantly a result of AM‐ester **3** accumulation. The spectral phasor analysis enabled the simultaneous detection of 9 relevant regions of interest based directly on a hyperspectral SRS image. Interestingly, a large number of cellular LDs were detected in the segments identified by (E) and (H), which is a likely reflection of the uptake of the fatty acid, stearic acid‐*d_35_* **5** during cellular steatosis.^[30]^ The ability to segment regions of co‐localised stearic acid‐*d_35_* **5** and AM‐ester **3** in (H) from regions that predominantly contain AM‐ester **3** (I) highlights the power of the approach particularly when these signals are overlapping in the Raman spectrum (Figure S6).

**Figure 4:**
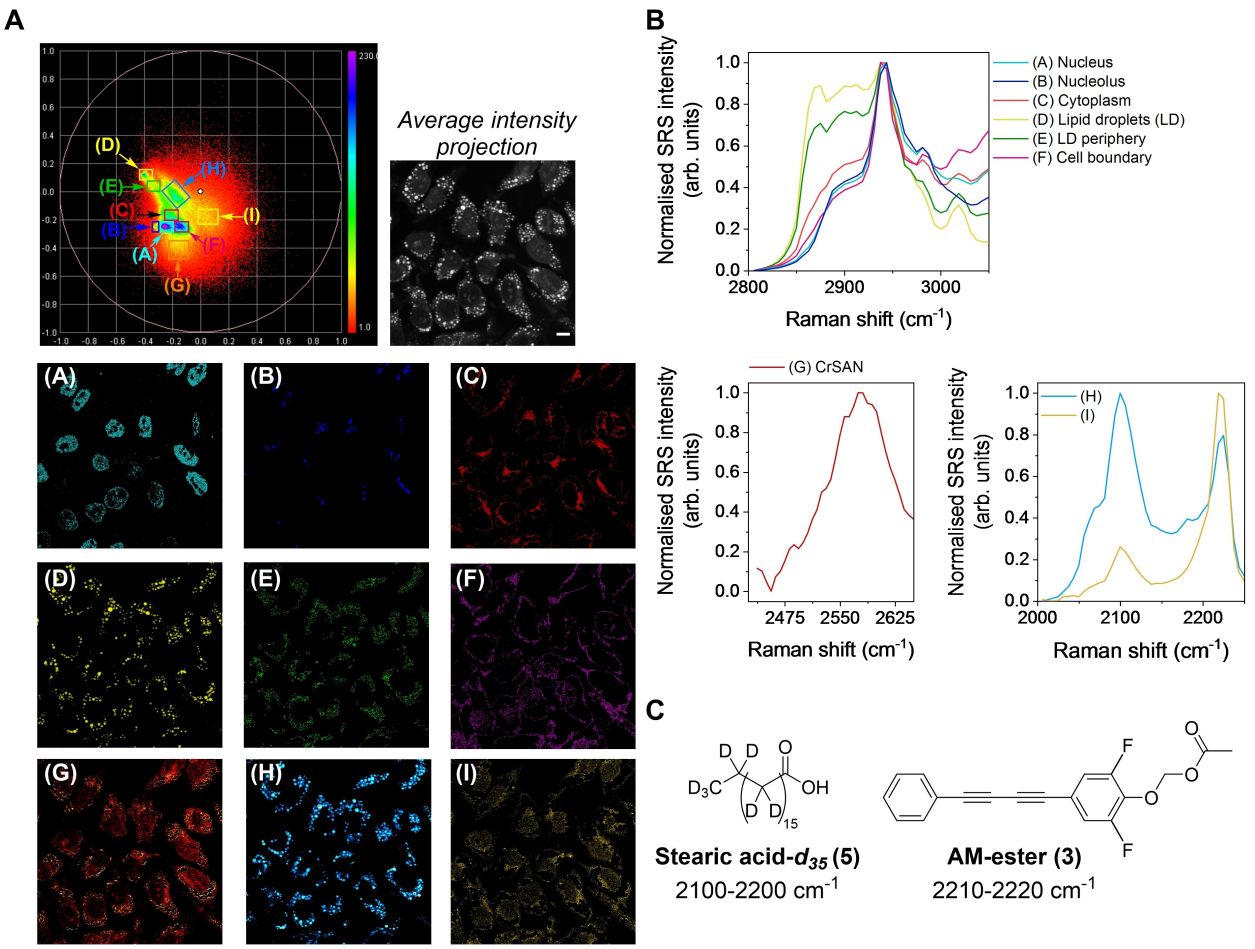
Multiplex imaging of metallacarboranes using spectral phasor analysis. A) HeLa cells were treated with stearic acid‐d_35_ **5** (200 μM, 8 h) before treatment with CrSAN (250 μM, 4 h) and AM‐ester **3** (100 μM, 30 min). SRS images were then acquired across the range 3050–2800 cm^−1^ (0.4 nm/~7 cm^−1^, 40 images), 2650–2450 cm^−1^ (0.4 nm/~7 cm^−1^, 35 images) and 2250–2000 cm^−1^ (0.4 nm/~7 cm^−1^, 40 images). Spectral phasor analysis was then performed on the combined image stacks. A) The spectral phasor has been segmented into the following areas as indicated by the coloured boxes and corresponding labels: (A) nucleus, (B) nucleolus, (C) cytoplasm, (D) lipid droplets (LDs), (E) LD periphery, (F) cell boundary, (G) CrSAN, (H) stearic acid‐d_35_ **5** and AM‐ester **3**, (I) AM‐ester. An average intensity projection is presented. Scale bar: 10 μM. B) Normalised average SRS spectra for the ROIs identified in (A). The spectra were normalised between 0–1. C) Chemical structures of stearic acid‐d_35_ **5** and AM‐ester **3** including the spectral range for their detection.

Given the hydrophobic nature of the AM‐ester **3** and the phenol that is produced following hydrolysis by intracellular esterases,^[20]^ it is unsurprising that the butadiyne is detected within cellular lipid droplets which are locally hydrophobic. To circumvent this, we investigated the cellular localisation of the more polar, unsymmetrical butadiyne, imidazole **4**, in combination with stearic acid‐d_35_ **5** and a metallacarborane (M=Fe, and Ni). HeLa cells were treated with stearic acid‐*d_35_* **5** (200 μM, 5 h) before washing and treating with FeSAN or NiSAN (250 μM, 4 h) and imidazole **4** (100 μM, 1 h). SRS images were acquired in a similar manner as per Figure 4. Spectral phasor analysis was performed on the resultant image stacks and the data are presented in Figure S7 (FeSAN) and Figure S8 (NiSAN). Cellular segmentation was performed as before, and the detection of the metallacarboranes was shown to be distinct from the regions of the spectral phasor plot containing the stearic acid‐*d_35_* **5** and imidazole **4**. Interestingly, the increased polarity of the imidazole **4** (cLogP=1.9) compared to AM‐ester **3** (cLogP=3.7), resulted in a pan‐cellular distribution detected at 2220 cm^−1^, including within the nuclei which is unusual for butadiyne motifs which have been largely detected within the cell cytoplasm.^[31]^ These results indicate further potential exists for modulating the Raman scattering properties and/or localisation of butadiynes through chemical functionalisation.

In summary, these studies demonstrate the unique ability of spectral phasor analysis to discriminate a triplex of bioorthogonal Raman reporters within the cellular environment. Coupled with imaging in the high wavenumber region, spectral phasor analysis has enabled 9‐colour detection. Our report highlights the potential of the metallacarboranes as Raman reporters with desirable properties for multiplex Raman imaging. Conjugation of *ortho*‐carborane has been reported as a method of Raman tagging,^[32]^ and we envisage similar opportunities for the metallacarboranes described here. Previous studies have set the scene for Raman imaging of B−H groups,[^13^, ^33^] and when coupled to SRS microscopy which offers faster imaging rates, our study demonstrated improved detection sensitivity at lower extracellular treatment concentrations and shorter incubation times, that resulted in different localisation patterns. We also highlight the potential in multiplex detection using different bioorthogonal groups that can be spectrally resolved using SRS and spectral phasor analysis. This new capability of increased bioorthogonality coupled with spectral phasor analysis offers significant opportunities for future cellular labelling and imaging studies.

## Conflict of interest

The authors declare no conflict of interest.

## Supporting information

As a service to our authors and readers, this journal provides supporting information supplied by the authors. Such materials are peer reviewed and may be re‐organized for online delivery, but are not copy‐edited or typeset. Technical support issues arising from supporting information (other than missing files) should be addressed to the authors.

Supporting Information

## Data Availability

The raw data supporting this research publication will be made available from the University of Strathclyde at the following link: 10.15129/0a0a96e1‐6fd4‐4e1e‐856f‐caa380bc1c14
