# Supplementary material for: Expanding the Range of Bioorthogonal Tags for Multiplex Stimulated Raman Scattering Microscopy
Source: Angew Chem Int Ed Engl. 2023 Oct 27;62(48):e202311530. doi: 10.1002/anie.202311530 (PMC10952743; doi:10.1002/anie.202311530)
Supplement: Supplementary file 1 — Supporting Information [file ANIE-62-0-s001.pdf]

Angewandte Chemie
Eine Zeitschrift der Gesellschaft Deutscher Chemiker

Supporting Information

Expanding the Range of Bioorthogonal Tags for Multiplex Stimulated Raman Scattering Microscopy

N. Murphy, W. J. Tipping, H. J. Braddick, L. T. Wilson, N. C. O. Tomkinson, K. Faulds, D. Graham, P. Farràs**

Contents

Materials and Methods……………………………………………………………………………2

Schemes S1-S3……………………………………………………………………………………4

Figures S1-S8……………………………………………………………………………………5

Table S1………………………………………………………………………………………………6

Table S2………………………………………………………………………………………………7

Synthetic procedures…………………………………………………………………………13

Figures S9-S19…………………………………………………………………………………14

References………………………………………………………………………………………25

Materials and Methods

Cell culture

HeLa cells were gifted from the Strathclyde Institute of Pharmacy and Biomedical Sciences (Glasgow) as a subculture from a stock received from the European Collection of Authenticated Cell Cultures (ECACC). HeLa cells were cultured in Dulbecco's Modified Eagle Medium (DMEM, glucose: 1 g/L) supplemented with 10% foetal bovine serum (FBS, Gibco™, Fisher Scientific), 1% penicillin/streptomycin (Gibco™, 10 000 U/mL, Fisher Scientific) and 1% Amphotericin B (Gibco™, 250 µg/mL, Fisher Scientific). Cells were maintained at 37 °C and 5% CO_2 in a humidified incubator and were routinely sub-cultured at *ca.* 80% confluency. The DMSO stock solutions were prepared fresh. Control samples were treated with DMSO (0.05% v/v) in culture medium.

cLogP

cLogP values were determined using ChemDraw Professional software.

SRS microscopy

An integrated laser system (picoEmerald™ S, Applied Physics & Electronics, Inc.) was used to produce two synchronised laser beams at 80 MHz repetition rate. A fundamental Stokes beam (1031.4 nm, 2 ps pulse width) was intensity modulated by an electro-optic-modulator (EoM) with >90% modulation depth, and a tunable pump beam (700–960 nm, 2 ps pulse width, <1 nm (10 cm^{-1}) spectral bandwidth) was produced by a built-in optical parametric oscillator. The pump and Stokes beams were spatially and temporally overlapped using two dichroic mirrors and a delay stage inside the laser system and coupled into an inverted laser-scanning microscope (Leica TCS SP8, Leica Microsystems) with optimised near-IR throughput. SRS images were acquired using 40× objective (HC PL IRAPO 40×, N.A. 1.10 water immersion lens) with a 9.75-48 µs pixel dwell time over a 512 × 512 or a 1024 × 1024 frame. The Stokes beam was modulated with a 20 MHz EoM. Forward scattered light was collected by a S1 N. A. 1.4 condenser lens (Leica Microsystems). Images were acquired at 12-bit image depth. The laser powers measured after the objective lens were in the range 10–30 mW for the pump beam only, 10–50 mW for the Stokes beam only and 20–70 mW (pump and Stokes beams). The spatial resolution of the system is ~450 nm (pump wavelength = 792 nm).

SRS imaging of live cells treated with metallacarboranes (Figure 2): HeLa cells were plated on high precision glass coverslips (#1.5H Thickness, 22 × 22 mm, Thorlabs) in a 6-well plate in DMEM at a concentration of 5 × 10^5 cells per mL and incubated at 37 °C and 5% CO_2 for a 24 h prior to treatment. Cells were treated with metallacarboranes from a 100 mM stock solution in DMSO (or DMSO as a control) and incubated at 37 °C and 5% CO_2 for the indicated time. Prior to imaging, the plates were aspirated and washed with PBS (2 × 2 mL). The coverslips were then affixed to glass microscope slides with a DMEM boundary between the glass layers prior to imaging. The images presented at 2570 cm^{-1} have been background subtracted using the images acquired at 2400 cm^{-1}. Look-up tables: 2930 cm^{-1} (greyscale, 0–4000 a.u.), 2851 cm^{-1} (red hot, 0–2500 a.u.) 2570 cm^{-1} (cyan hot, 100–2000 a.u.).

SRS imaging and spectral phasor analysis (Figure 3 and 4): HeLa cells were plated on high precision glass coverslips (#1.5H Thickness, 22 × 22 mm, Thorlabs) in a 6-well plate in DMEM at a concentration of 5 × 10^5 cells per mL and incubated at 37 °C and 5% CO_2 for a 24 h prior to treatment. Cells were treated with metallacarboranes from a 100 mM stock solution in DMSO (or DMSO as a control) and incubated at 37 °C and 5% CO_2 for the indicated time. Prior to imaging, the plates were aspirated and washed with PBS (2 × 2 mL). The cells were fixed with paraformaldehyde (4% in PBS, 15 min at rt) and washed with PBS (2 × 2 mL). The

coverslips were then affixed to glass microscope slides with a PBS boundary between the glass layers prior to imaging.

Given the differences in the Raman scattering cross section and relative intensities compared to EdU (RIE) of C–D, C≡C and B–H bonds, we found it necessary to use different treatment concentrations (and times) in order to achieve a detectable intracellular signal for each probe.

Spectral phasor analysis: The SRS image data set across the range 2800-3050 cm^{-1} was imported into ImageJ and an average intensity projection was created. The spectral phasor analysis was performed as described by Fu *et al. Anal. Chem.*, **2014**, *86*, 4115-4119. Segmentation of the phasor plot was performed manually using regions of interest (ROIs) to create images of discrete cellular locations. The corresponding average spectra for each ROI is plotted using Origin.

Cell viability assay: HeLa cells (5000 cells/well, 100 µL) were plated in a 96-well plate and incubated overnight. The DMEM culture media was removed by gentle aspiration and replaced with media containing DMSO (0.05% v/v control), Triton X-100 (0.05% v/v positive control), CoSAN (250 µM, or 400 µM for 4h) or CoSAN (1 mM, 15 min). The media was aspirated after the desired timepoint, replaced with DMEM (100 µL) and treated with AlamarBlue reagent (10 µL) for 4 h at 37 °C. A minimum of 6 wells were analysed per treatment in two biological repeats. The plates were analysed using a Tecan Spark plate reader with absorbance measured at 570 nm. Data represent the mean %viability ±S.D. The %viability was determined using the following formula:

Cell viability (%) = $(A_{sample} - A_{TritonX})/(A_{DMSO} - A_{TritonX})*100$

Data analysis of SRS images

False colour assignments, scale bars and image overlays were added to images using ImageJ software. Consistent brightness and contrast settings were used when comparing image datasets.

Raman spectroscopy

Raman spectra were acquired on a Renishaw InVia Raman microscope equipped with a 532 nm Nd:YAG laser providing a maximum power of 45 mW using a 1800 l/mm grating, a 633 nm HeNe laser providing a maximum power of 17 mW using a 1200 l/mm grating, and a 785 nm diode laser providing a maximum power of 300 mW using a 1200 l/mm grating.

Neat samples: A microgram quantity of solid material was transferred to a CaF_2 disc and imaged directly using the imaging conditions as outlined in the corresponding Figure legend.

Cell samples: HeLa cells were plated directly onto a CaF_2 disc at a concentration of 1×10^5 cells/mL in DMEM media. The cells were cultured for 24h prior to treating with stearic acid-d_{35} (200 µM, 8h) before washing and secondary treatment with CrSAN (250 µM, 4 h) and AM-ester **3** (100 µM, 30 min). Raman spectra were acquired using 532 nm excitation with a 60× lens (36 mW) for 10 s.

Data processing for Raman spectra: All spectra were processed in WiRE 4.4™ software enabling cosmic ray removal and baseline subtraction. Peak normalisation was performed in OriginPro2018 software and the peak areas determined using the Integrate tool.

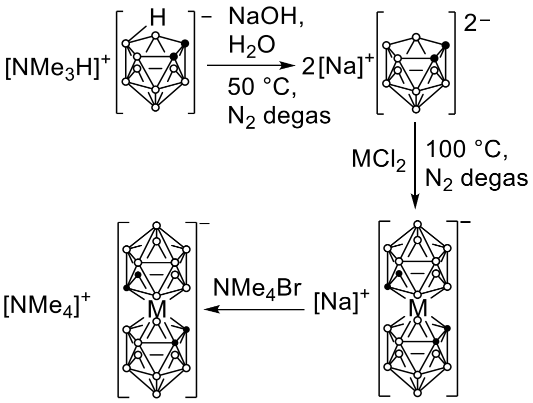

Scheme S1 Preparation of Co, Fe, Ni metallacarboranes.

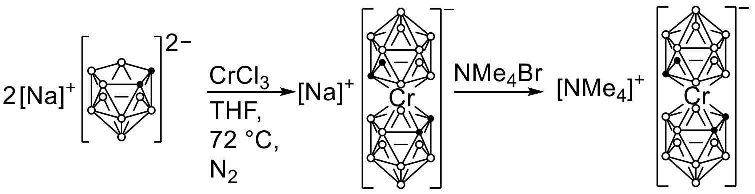

Scheme S2 Procedure for synthesis of Cr metallacarborane.

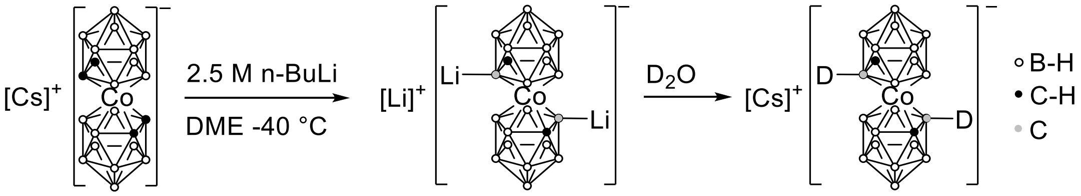

Scheme S3 Procedure for synthesis of deuterated cobalt (III) *bis* dicarbollide.

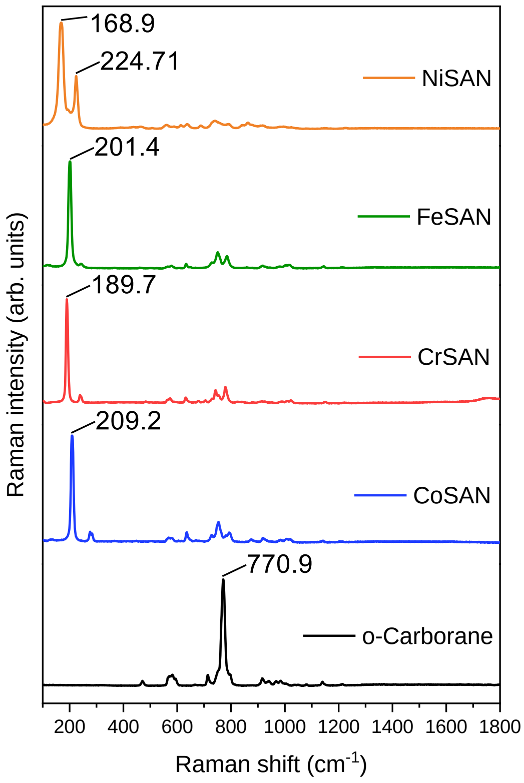

Figure S1 Fingerprint region Raman spectra of carboranes. Raman spectra of *ortho*-carborane and the metallacarboranes were acquired in solid form using 785 nm excitation with a 20× lens (~20 mW) for 10 s. The spectra are normalised (0-1) and offset for clarity. Peak assignments are in cm^{-1}. The spectra are a portion of the same acquisition as those presented in **Figure 1C** that represent the high wavenumber region of the Raman spectrum containing the B–H vibrational modes (2400-3200 cm^{-1}).

Table S1 Peak assignments for the Raman spectra acquired from *ortho*-carborane and metallacarboranes (M=Co, Cr, Fe, Ni).

**Raman shift (cm^{-1})**					
***Ortho*-Carborane**	**CoSAN**	**CrSAN**	**FeSAN**	**NiSAN**	**Assignment**
	209.2 (vs)	189.7 (vs)	201.4 (vs)	168.9 (vs)	Whole molecule stretching[a]
	276.1 (s)	238.9 (s)	242.8 (s)	224.7 (s)	Carborane cages rocking[a]
471.2					
582.6, 594.7	586.0	574.1	562.2, 580.2	559.5, 614.0	
	635.7	632.1	633.3	638.1	B-B-M bending[a]
714.4				687.1	
	728.6	729.8	728.6		
	753.3	742.7	751.0	739.2	Icosahedral breathing mode[a]
770.9	793.1	779.1	786.1	, 793.1	
	875.3	881.1		840.8, 862.7	
916.6	917.7	915.5	918.9	916.6	
941.6	985.7	988.0	981.2	983.5	
967.7	1005.7	1008.2	1001.5,	995.9	
1033.9	1019.4	1022.8	1020.5	1023.9	
1082.8					
1140.0, 1214.9	1142.2	1152.0	1145.5	1147.7, 1227.9	δ(CH)[b]
2581.7, 2615.6, 2633.2	2535.3, 2554.9, 2572.8, 2586.6, 2598.7, 2621.2	2503.3, 2537.8, 2550.0, 2567.9, 2589.8, 2603.5, 2634.0	2552.7, 2568.9, 2582.6,	2534.5, 2580.1, 2609.1, 2634.8	B-H stretching[a,b]
3073.9	3031.9, 3042.1, 3047.9	3010.8, 3018.8, 3033.4	3022.1, 3030.6, 3042.6	3057.3, 3063.8	C-H stretching[a,b]

Notes: where no assignment has been made, Raman spectral frequencies have been grouped based on similar wavenumber positions. Most vibrations having frequencies in the region from 450 to 1100 cm^{-1} are of complex origin, and therefore challenges remain over their interpretation and assignment. References: [a] B. Barszcz *et al. J. Mol. Struct.*, **2010**, *976*, 196-199; [b] L. A. Leites, *Chem. Rev.*, **1992**, *92*, 279-323. (vs) very strong, (s) strong.

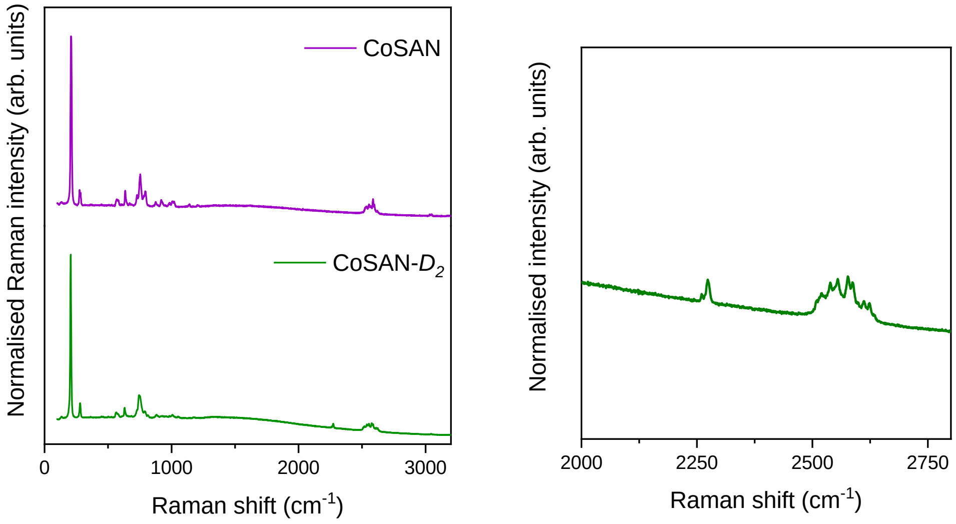

Figure S2 Raman spectrum of CoSAN and CoSAN-*D*2*.* Raman spectra of the CoSANs were acquired in solid form using 785 nm excitation with a 20× lens (~20 mW) for 10 s. The spectra are normalised (0-1) and offset for clarity. An expanded view of CoSAN-D_2 is provided.

Table S2 Peak assignment for CoSAN and CoSAN-*D*2.

**CoSAN**	**CoSAN-D_2**	**Assignment**
209.2 (vs)	206.6 (vs)	Whole molecule stretching[a]
276.1 (s)	280.0 (s)	Carborane cages rocking[a]
586.0	565.6, 589.9	
635.7	630.9	B-B-M bending[a]
728.6	722.7	
753.3	743.9	Icosahedral breathing mode[a]
793.1	790.8	
875.3	881.1	
917.7	918.9	
985.7	983.5	
1005.7	1007.1	
1019.4	1051.8	
1142.2	1171.6	δ(CH)[b]
	2260.4, 2273.4	C-D
2535.3, 2554.9, 2572.8, 2586.6, 2598.7, 2621.2	2518.9, 2538.6, 2554.9, 2576.8, 2588.2, 2599.5, 2611.5, 2623.6	B-H
3031.9, 3042.1, 3047.9	3024.7, 3045.7	C-H

Notes: where no assignment has been made, Raman spectral frequencies have been grouped based on similar wavenumber positions. Most vibrations having frequencies in the region from 450 to 1100 cm^{-1} are of complex origin, and therefore challenges remain over their interpretation and assignment. References: [a] B. Barszcz *et al. J. Mol. Struct.*, **2010**, *976*, 196-199; [b] L. A. Leites, *Chem. Rev.*, **1992**, *92*, 279-323. (vs) very strong, (s) strong.

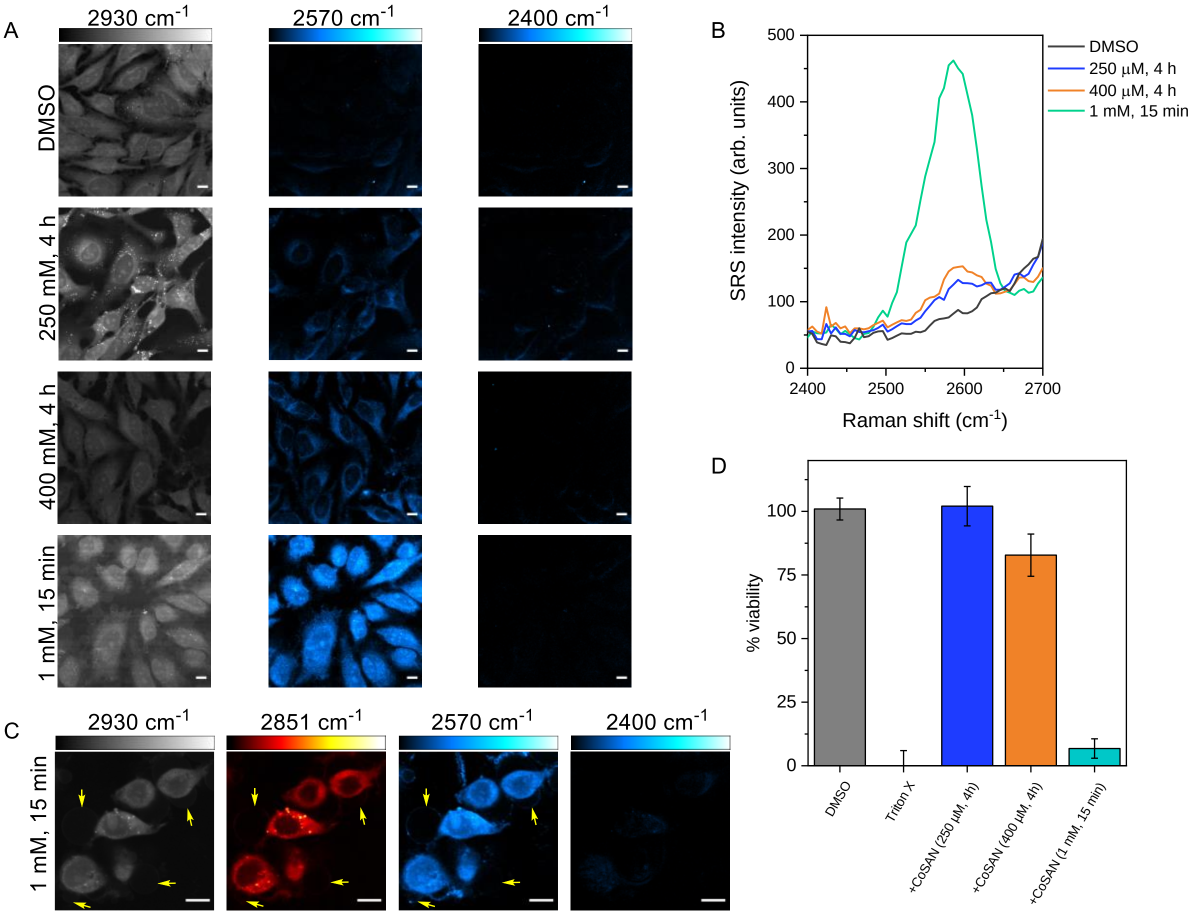

Figure S3 A concentration study of metallacarborane uptake. **A** SRS imaging of live HeLa cells treated with DMSO (control), CoSAN (250 µM or 400 µM for 4 h) or CoSAN (1 mM, 15 min). SRS images acquired at 2930 cm^{-1} (CH_3, proteins), 2570 cm^{-1} (B-H, CoSAN) and 2400 cm^{-1} (off-resonance). Scale bars: 10 µm. **B** SRS spectra from the cells presented in **A** across the range 2400-2700 cm^{-1}. **C** Replicate analysis of HeLa cells treated with CoSAN (1 mM, 15 min) and SRS images were acquired from live cells at the following frequencies: 2930 cm^{-1} (CH_3, proteins), 2851 cm^{-1} (CH_2, lipids), 2570 cm^{-1} (B-H) and 2400 cm^{-1} (off-resonance). Yellow arrowheads indicate membrane blebbing. Scale bars: 10 µm. **D** Cell viability assessment of HeLa cells treated with DMSO (control), Triton X-100 (positive control) and CoSAN at the indicated timepoints and concentrations as per **A**. Data represent the mean %viability relative to DMSO (100%) with error bars: ±S.D.

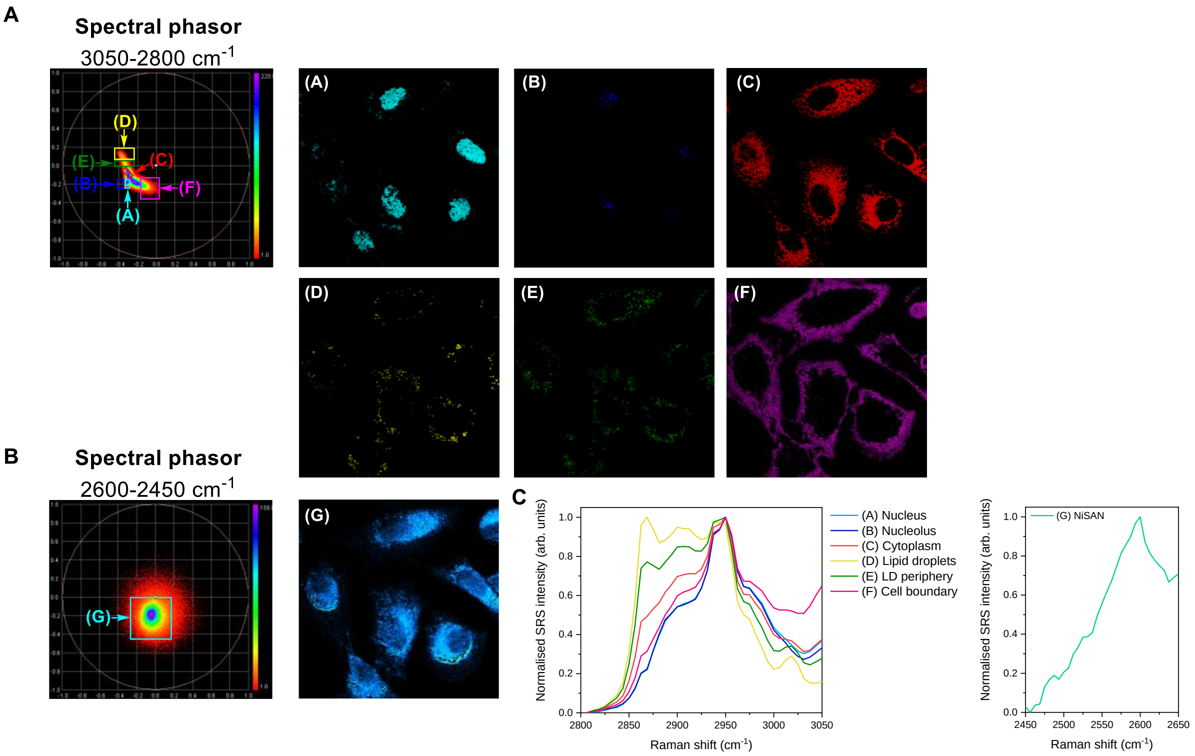

Figure S4 Cellular segmentation using spectral phasor analysis. HeLa cells were treated with NiSAN (500 µM, 15 min) before SRS images were acquired across the range **A** 3050-2800 cm^{-1} (0.4 nm, 40 images) and **B** 2600-2450 cm^{-1} (0.4 nm, 35 images). The spectral phasor plots have been segmented into the following regions: (A) nucleus, (B) nucleolus, (C) cytoplasm, (D) lipid droplets, (E) lipid droplet periphery, (F) cell boundary and (G) NiSAN. **C** Normalised SRS spectra corresponding to the segments (A) – (G). Spectra have been normalised between 0-1.

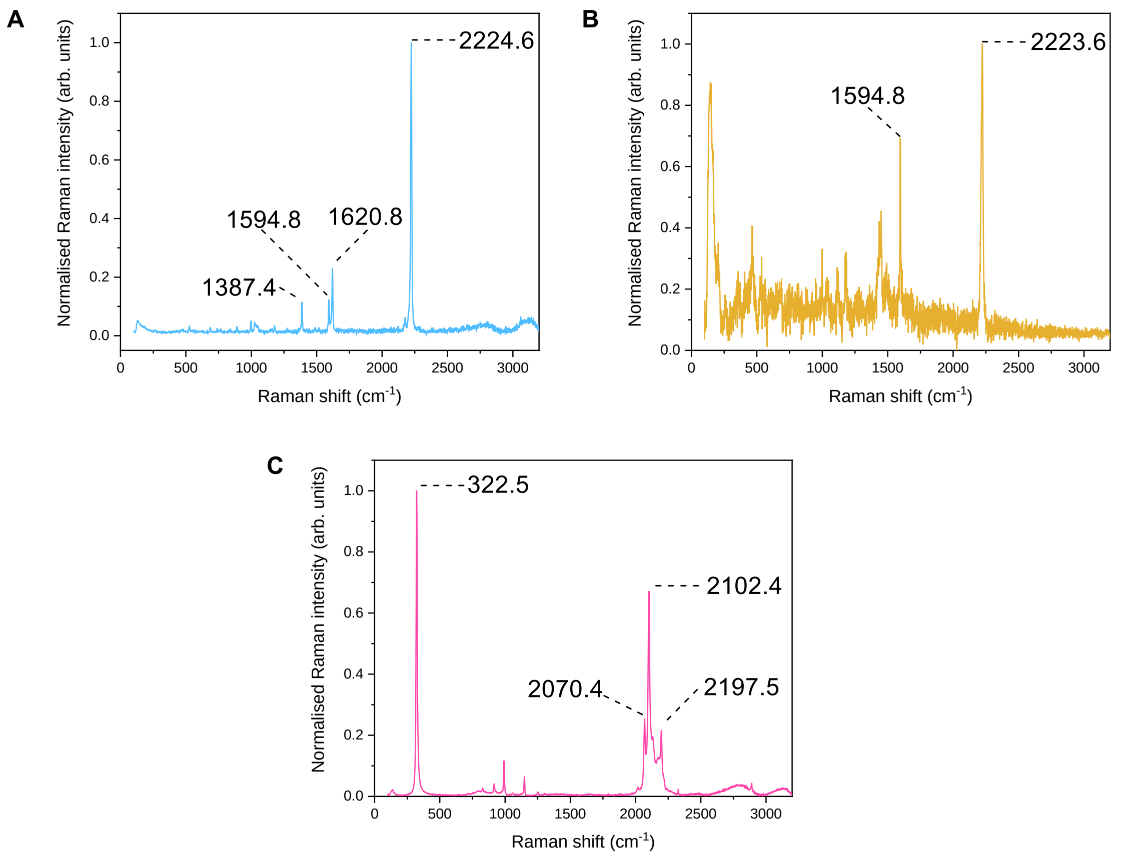

Figure S5 Raman spectra of the butadiynes **3** & **4** and stearic acid-*d*$_{35}$ used in this study. **A** Raman spectrum of AM-ester **3** acquired using 532 nm excitation with a 20× lens (0.36 mW) for 10 s, **B** Raman spectrum of imidazole **4** using 785 nm excitation with a 20× lens (0.2 mW) for 10 s and **C** Raman spectrum of stearic acid-*d*$_{35}$ **5** using 532 nm excitation with a 20× lens (36 mW) for 10 s. Peak assignments in cm^{-1}.

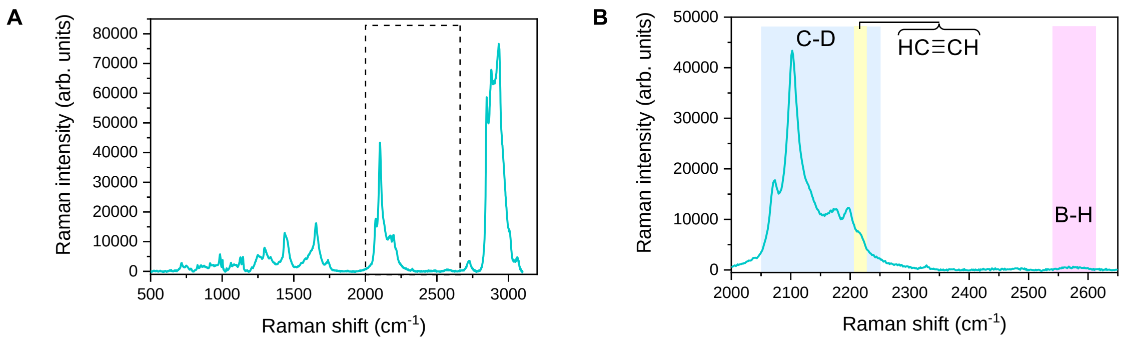

Figure S6 Detection of a triplex of bio-orthogonal Raman groups using Raman spectroscopy. Average Raman spectrum from a HeLa cell treated with stearic acid-*d*$_{35}$ **5** (200 µM, 8h) before washing and secondary treatment with CrSAN (250 µM, 4 h) and AM-ester **3** (100 µM, 30 min). **A** Average Raman spectrum from a single cell. **B** Expanded view of the region 2000-2650 cm^{-1} showing the overlapping C-D and C≡C peaks together with the metallacarborane B-H stretches. Raman spectrum acquired using 532 nm excitation with a 60× lens (36 mW) for 10 s.

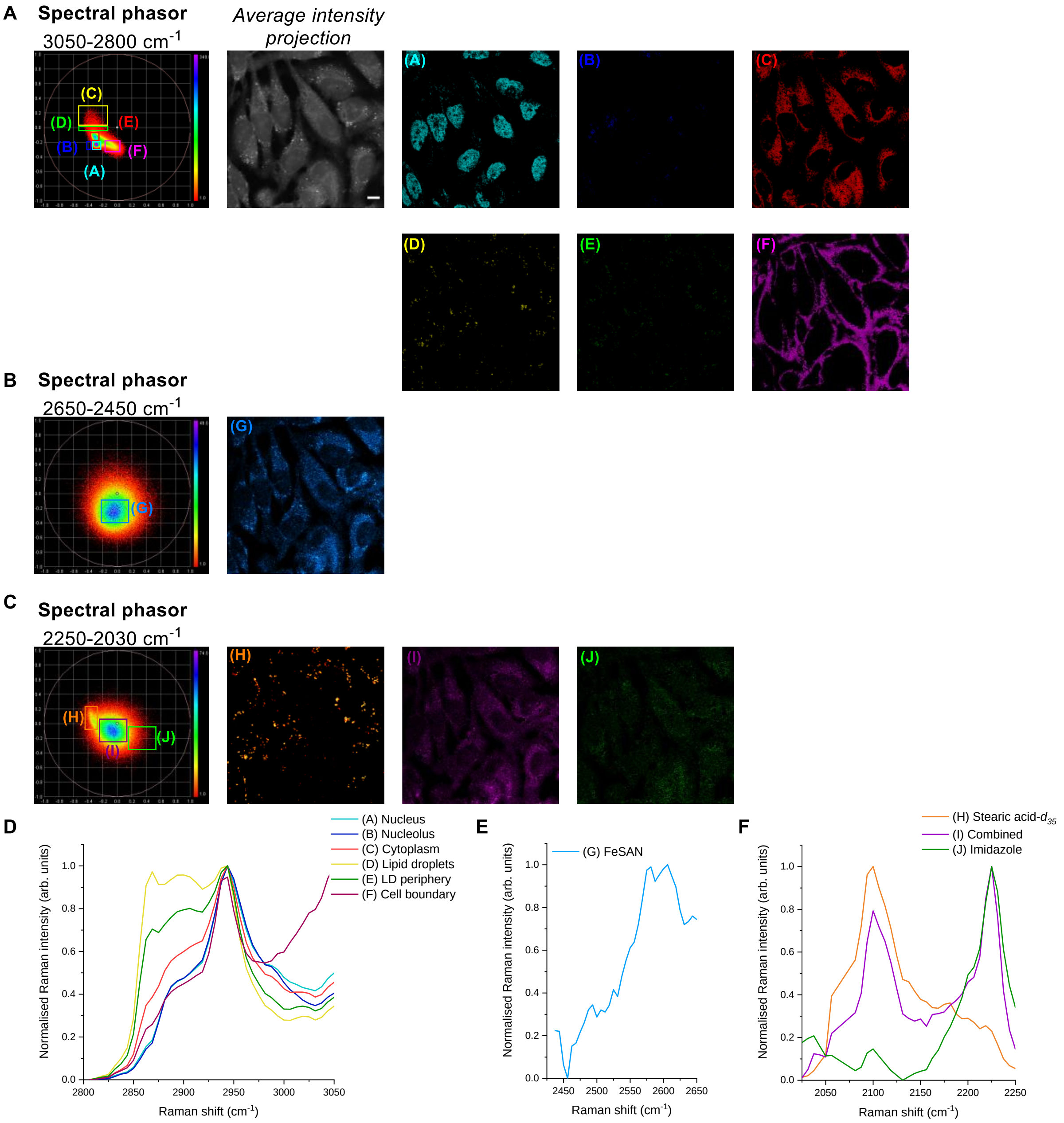

Figure S7 Spectral unmixing of a triplex mixture of bio-orthogonal Raman peaks. HeLa cells were treated with stearic acid-*d*$_{35}$ **5** (200 μM, 5h) before treatment with FeSAN (250 μM, 4h) and imidazole **4** (100 μM, 1 h). The cells were imaged using SRS microscopy across the ranges: **A** 3050-2800 cm^{-1} (0.4 nm, 40 images), **B** 2650-2450 cm^{-1} (0.4 nm, 35 images) and **C** 2250-2030 cm^{-1} (0.4 nm, 40 images). Scale bar: 10 μm. Colour-coded segmentation of the spectral phasor plots are presented in the segmented images labelled (A)-(J). **D-F** Normalised SRS spectra of the segmented regions (A)-(J).

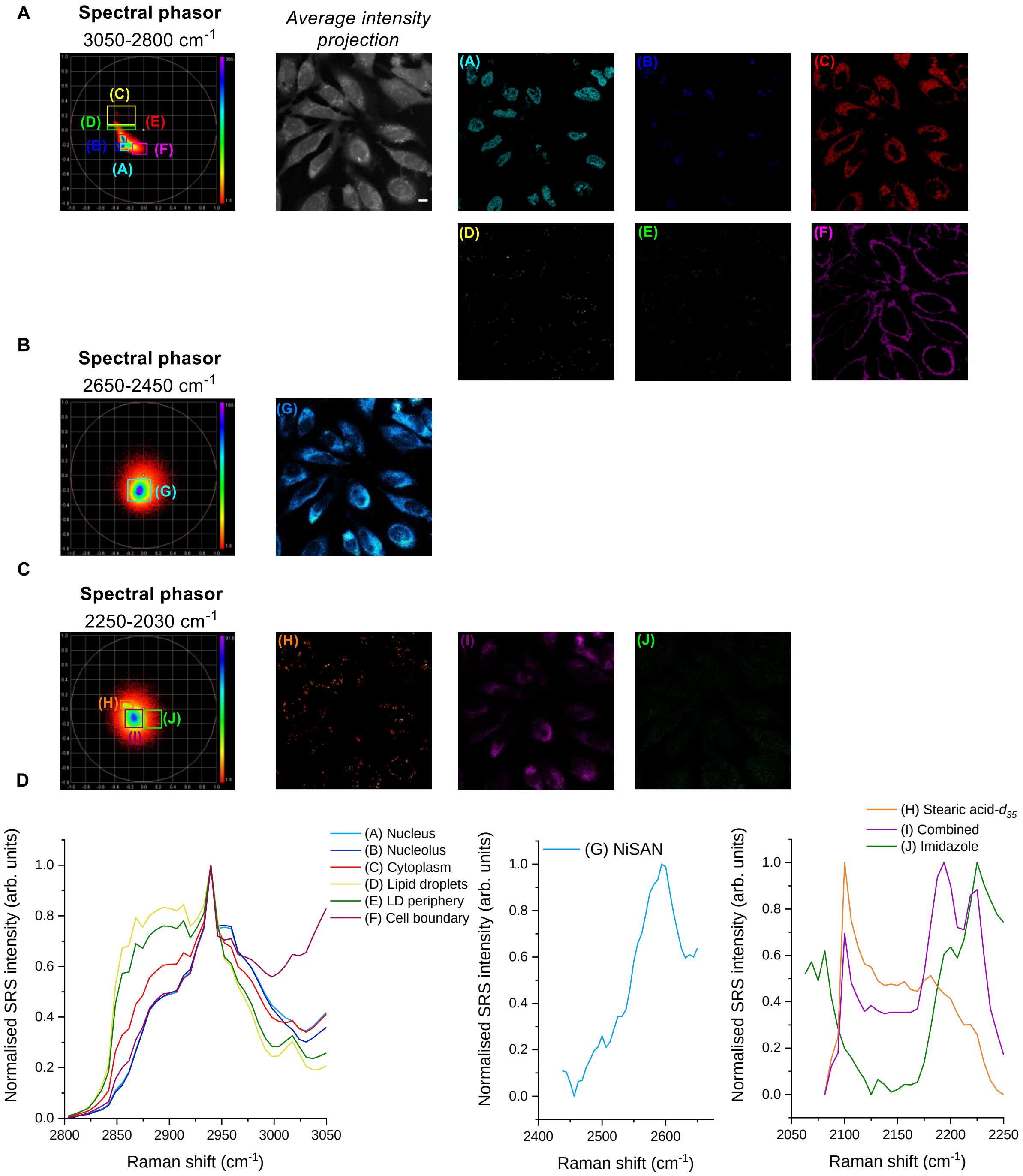

Figure S8 Spectral unmixing of a triplex mixture of bio-orthogonal Raman peaks. HeLa cells were treated with stearic acid-*d*$_{35}$ **5** (200 µM, 5h) before treatment with NiSAN (250 µM, 4h) and imidazole **4** (100 µM, 1 h). The cells were imaged using SRS microscopy across the ranges: **A** 3050-2800 cm^{-1} (0.4 nm, 40 images), **B** 2650-2450 cm^{-1} (0.4 nm, 35 images) and **C** 2250-2030 cm^{-1} (0.4 nm, 40 images). Scale bar: 10 µm. The colour-coded segmentation of the spectral phasor plots are presented in the segmented images labelled (A)-(J). **D** Normalised SRS spectra of the segmented regions (A)-(J).

Experimental: synthetic procedures

^{1}H, ^{11}B and ^{13}C NMR spectroscopy was performed on a Varian 500 MHZ 54 mm AR spectrometer. The spectra for ^{1}H, ^{13}C, and ^{11}B were recorded at 500 MHz, 125 MHz and 160.4 MHz respectively. Trimethylsilane was used as a reference for ^{1}H and ^{13}C, with boron trifluoride diethyl etherate used for ^{11}B. Deuterated solvents used included D_2O, C_2D_3N. Solvent stated on spectra in supporting information.

Experiments were carried out, except when noted, under a dry, oxygen-free dinitrogen atmosphere using standard Schlenk techniques, with some subsequent manipulation in the open laboratory. *o*-$C_2B_{10}H_{12}$ and [Cs][CoSAN] were purchased from Katchem and used as received. All other solvents and organic and inorganic salts were purchased from Across Organics, TCI or Sigma-Aldrich at analytical reagent grade and were used as received. Anhydrous THF was obtained by passing HPLC grade THF through a Grubbs system under inert atmosphere.

Mass spectra were attained using an Agilent 6510 QTOF LC/MS/MS system. Ionization was achieved by electrospray ionization in negative ion mode (ESI−). The capillary voltage was set to 2.5 kV. The cone temperature was 300 °C and the source temperature was 350 °C.

Preparation of cobalt (III) bis-dicarbollide [Na][CoSAN]

CoSAN was prepared following a procedure reported in the literature.[1] A solution of $[NMe_3H][C_2B_9H_{12}]$ (100 mg, 0.52 µmol) in an aqueous 40% NaOH solution (1 mL) was purged with nitrogen to remove released trimethylamine. To this of $CoCl_2$ (109.7 mg, 0.845 µmol) in an aqueous 40% NaOH solution (1 mL) was added, and the solution heated to reflux at 110 °C for 15 mins. This was then diluted with distilled water (5 mL), allowed to cool to room temperature and filtered. Extractions were carried out with filtrate/diethyl ether (3 x 10 mL) and the organic layer retained. The solvent was evaporated, and the residue taken up in distilled water (4 mL). To this a concentrated solution of tetramethylammonium bromide was added, yielding a yellow precipitate. This was filtered, dried *in vacuo* to give $[NMe_4]$[CoSAN] (72.41 mg, 182 µmol, 70%). ^{11}B NMR matched signals described in the literature.[1] The sodium salt prepared as described in the literature.[2]

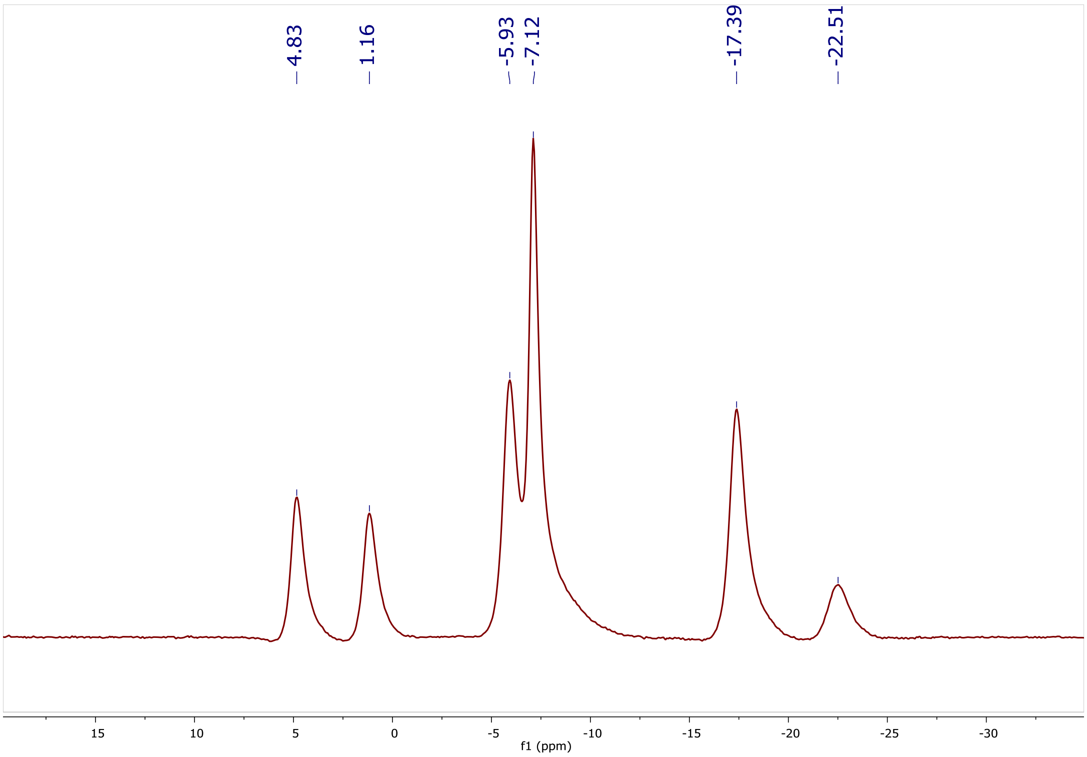

Figure S9 $^{11}B\{^{1}H\}$ NMR spectrum of [Na][CoSAN] in D_2O.

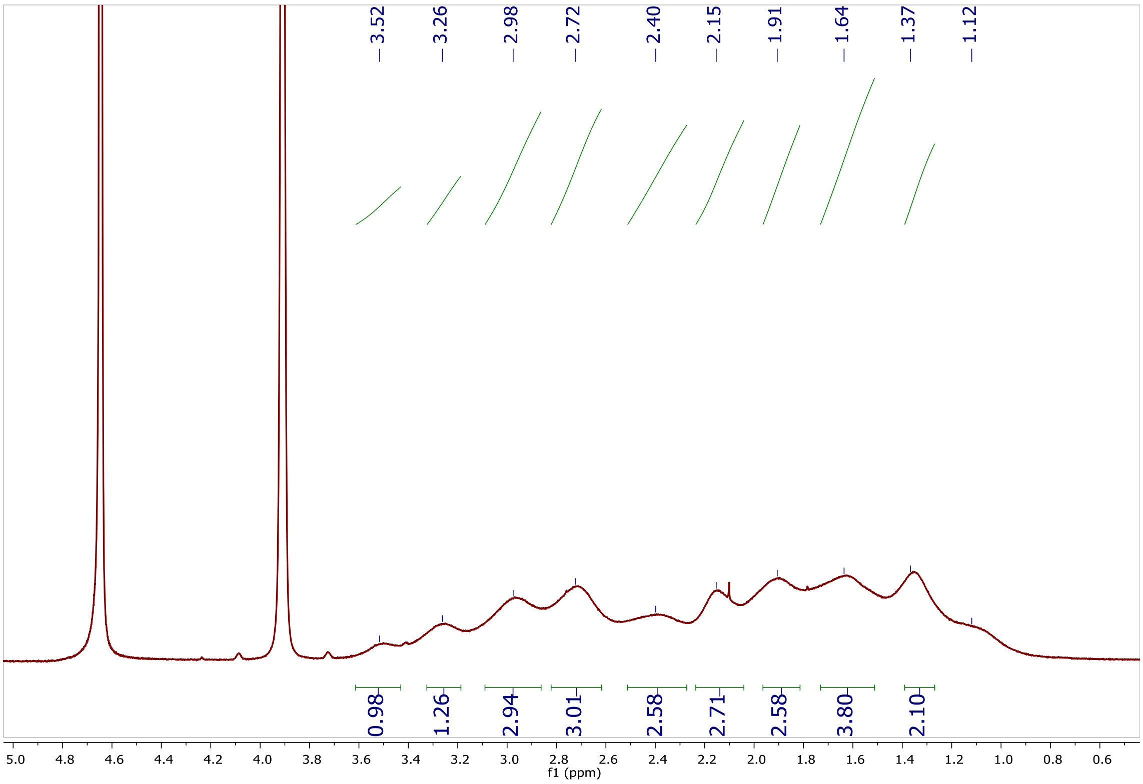

Figure S10 ^{1}H NMR spectrum of [Na][CoSAN] in D_2O.

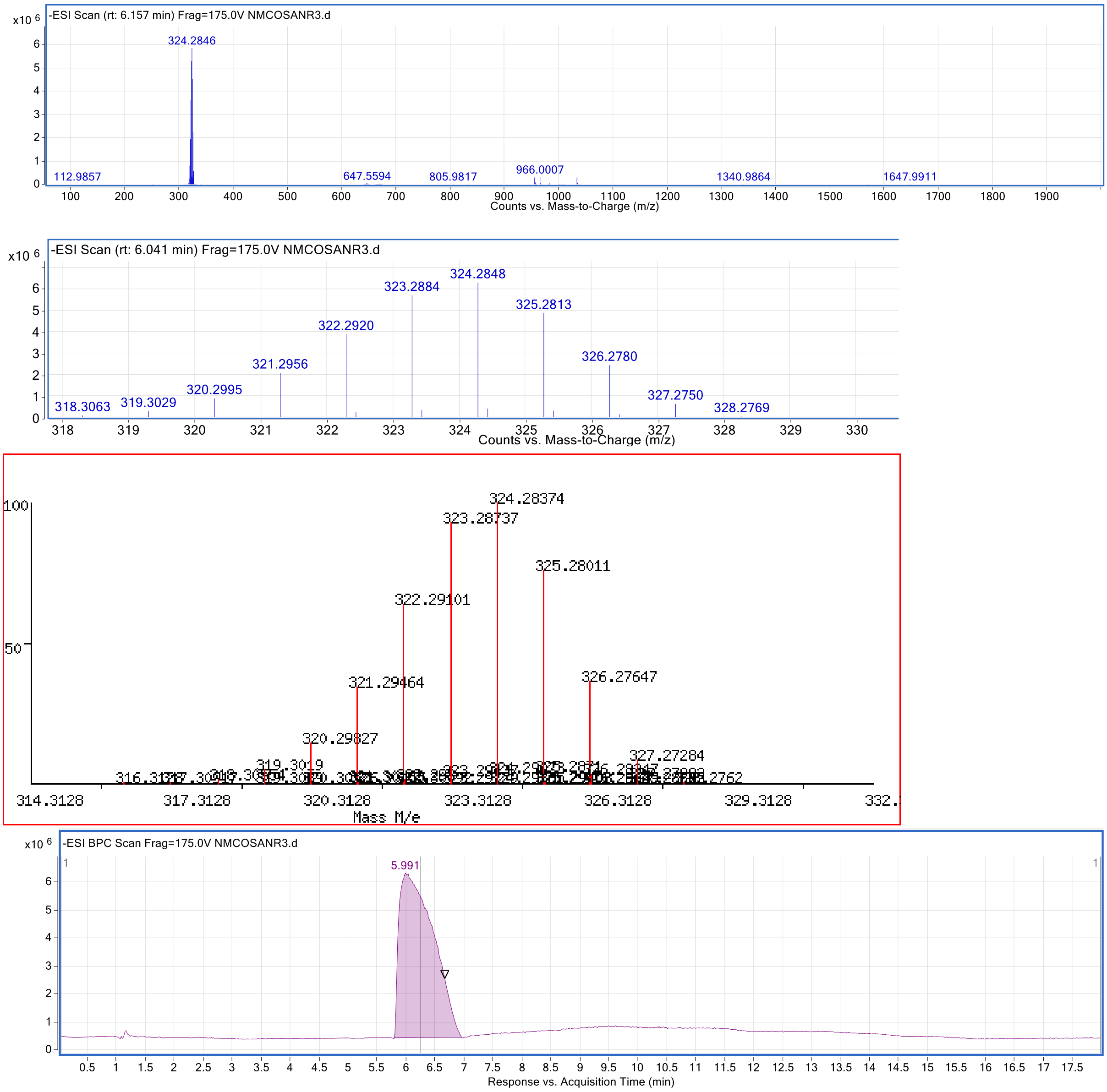

Figure S11 BPC scan and ESI peaks of [Na][CoSAN] (324.2848 m/z)

Preparation of iron (III) bis-dicarbollide [Na][FeSAN]

FeSAN was prepared following a procedure reported in the literature.[1] A solution of $[NMe_3H][C_2B_9H_{12}]$ (100 mg, 0.52 µmol) in an aqueous 40% NaOH solution (1 mL) was purged with nitrogen to remove released trimethylamine. To this $FeCl_2•4H_2O$ (218.69 mg,1.11 mmol) in an aqueous 40% NaOH solution (1 mL) was added, and the solution heated to reflux at 110 °C for 15 mins. This was then diluted with distilled water (5 mL), allowed to cool to room temperature and filtered. Extractions were carried out with filtrate/diethyl ether (3 x 10 mL) and the organic layer retained. The solvent was evaporated, and the residue taken up in distilled water (4 mL). To this a concentrated solution of tetramethylammonium bromide was added, yielding a black-red precipitate. This was filtered, dried *in vacuo* to give $[NMe_4][FeSAN]^-$

(48.25 mg, 122.2 µmol, 47%), ^{11}B NMR matched signals described in the literature.[1] The sodium salt was prepared as described in the literature.[2]

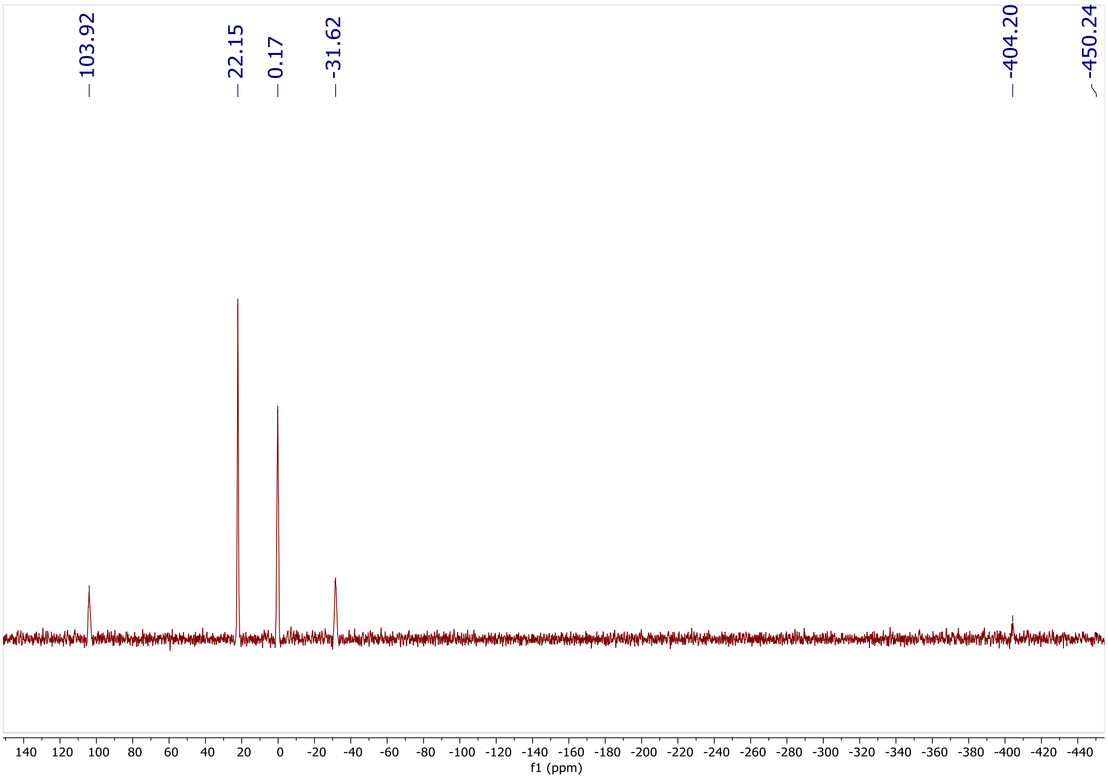

Figure S12 $^{11}B\{^{1}H\}$ NMR spectrum of [Na][FeSAN] in D_2O.

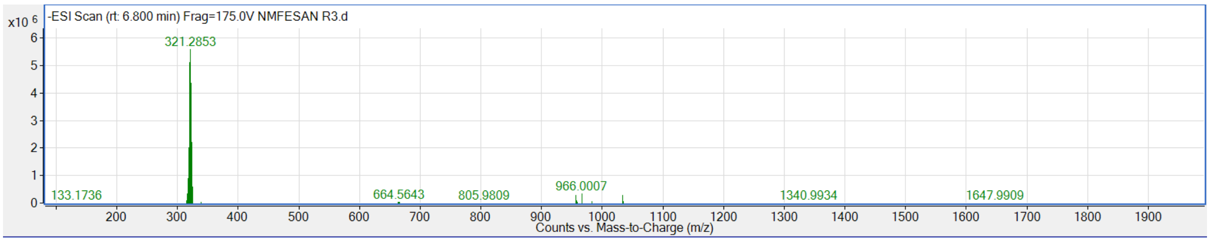

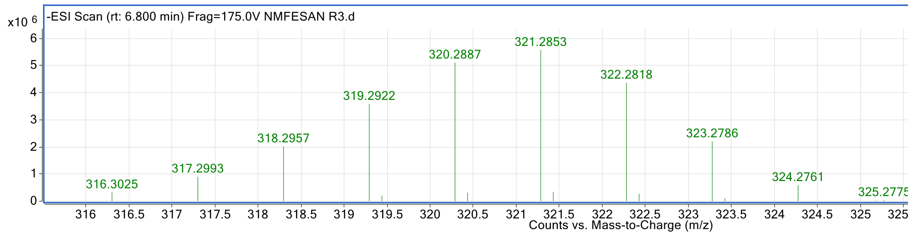

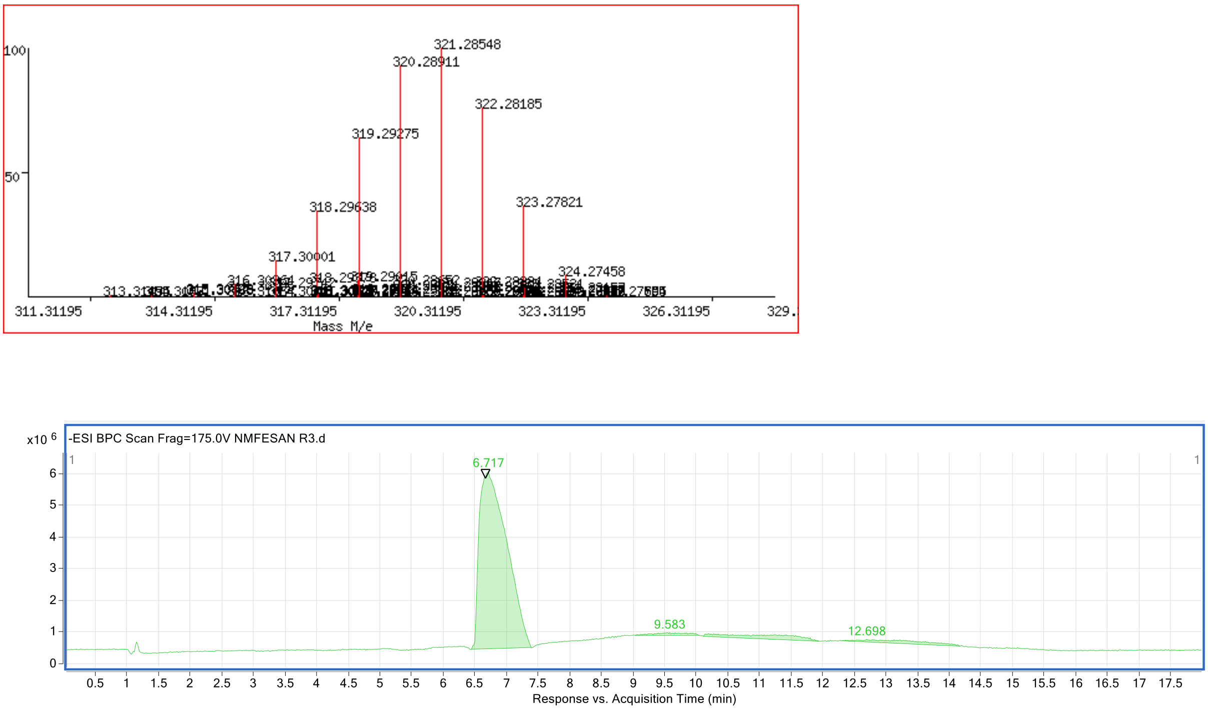

Figure S13 BPC scan and ESI peaks of [Na][FeSAN] (321.2853 m/z)

Preparation of nickel (III) bis-dicarbollide [Na][NiSAN]

NiSAN was prepared following a procedure reported in the literature.[1] A solution of $[NMe_3H][C_2B_9H_{12}]$ (100 mg, 0.52 µmol) prepared as described in the literature in an aqueous 40% NaOH solution (1 mL) was purged with nitrogen to remove released trimethylamine. To this a $NiCl_2.6H_2O$ (109.17 mg, 0.84 µmol) solution in 40% NaOH (1 mL) was added, and the solution heated to reflux at 110 °C for 15 mins. This was then diluted with distilled water (5 mL), allowed to cool to room temperature and filtered. Extractions were carried out with filtrate/diethyl ether (3 x 10 mL) and the organic layer retained. The solvent was evaporated, and the residue taken up in distilled water (4 mL). To this a concentrated solution of tetramethylammonium bromide was added, yielding a yellow-orange precipitate. This was filtered and dried *in vacuo* to give $[NMe_4]$[NiSAN] (66.13 mg, 166.4 µmol, 64%), ^{11}B NMR matched signals described in the literature.[1] The sodium salt was prepared as described in the literature.[2]

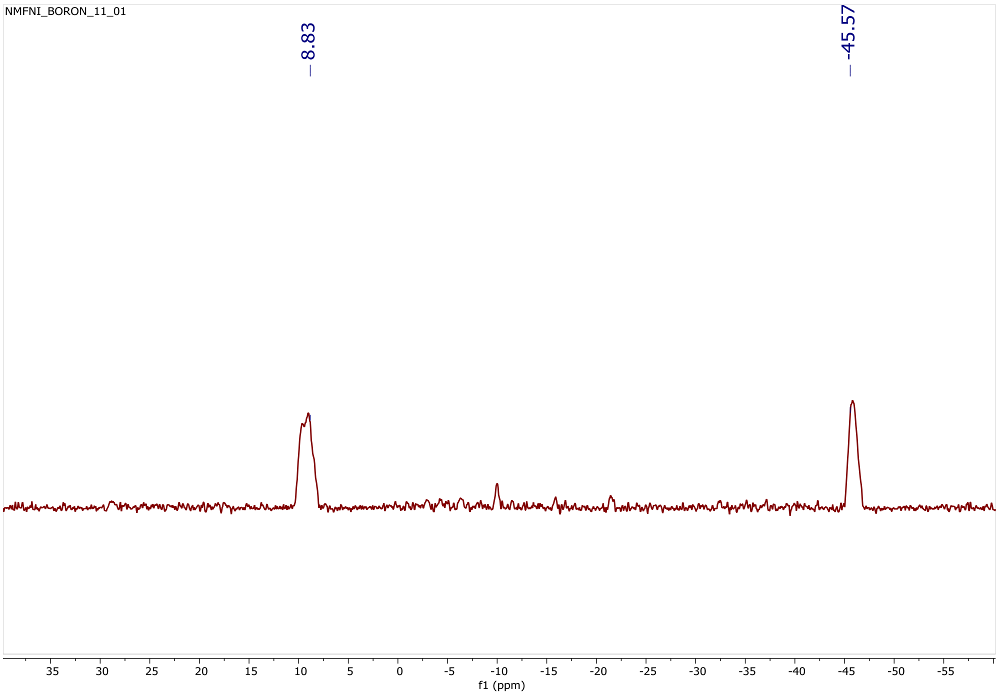

Figure S14 $^{11}B\{^{1}H\}$ NMR spectrum of [Na][NiSAN] in D_2O.

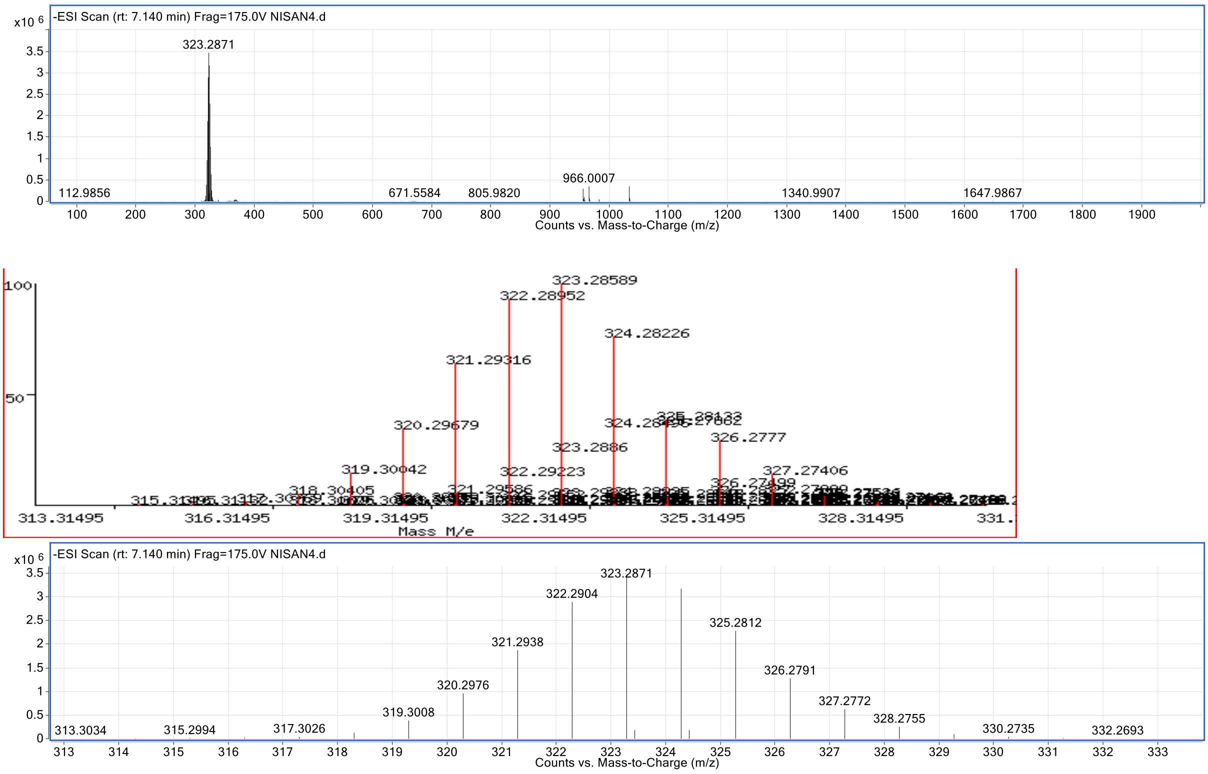

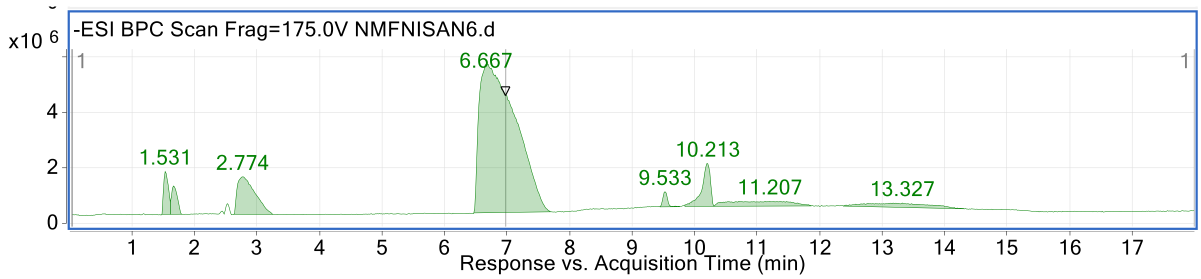

Figure S15 Mass spectrum of [Na][NISAN] (top) matching predicted distribution (bottom) (323.2884 m/z).

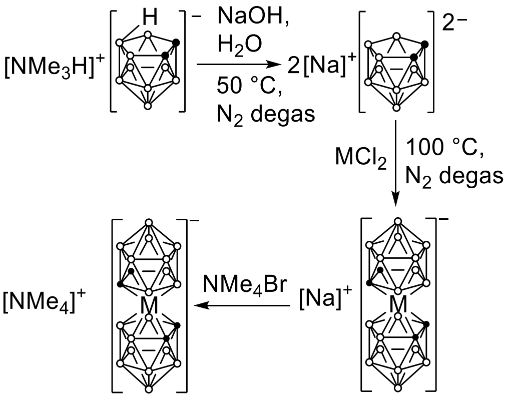

Scheme S1 Preparation of Co, Fe, Ni metallacarboranes.

Preparation of chromium (III) bis-dicarbollide [Na][CrSAN]

CrSAN was prepared following a procedure reported in the literature.[3] $CrCl_3$ (39.73 mg, 0.25 mmol) in anhydrous THF (0.5 mL) was added via degassed syringe to a solution of 2[Na][$C_2B_9H_{11}$] (88.6mg, 0.5 mmol) (prepared as described in the literature)[1] in anhydrous THF (0.5 mL). This was heated at 72 °C to reflux for 3 h under inert atmosphere. The solvent was evaporated under reduced pressure. Extractions were carried out with filtrate/diethyl ether (3 x 10 mL) and the organic layer retained. The solvent was evaporated, and the residue taken up in distilled water (4 mL). To this a concentrated solution of tetramethylammonium bromide was added, yielding a purple precipitate. This was filtered, washed with distilled water and dried *in vacuo* to give [NMe_4][CrSAN] (62 mg, 158.6 μmol, 61%), ^{11}B NMR matched signals described literature.[3] The sodium salt was prepared as described in the literature.[2]

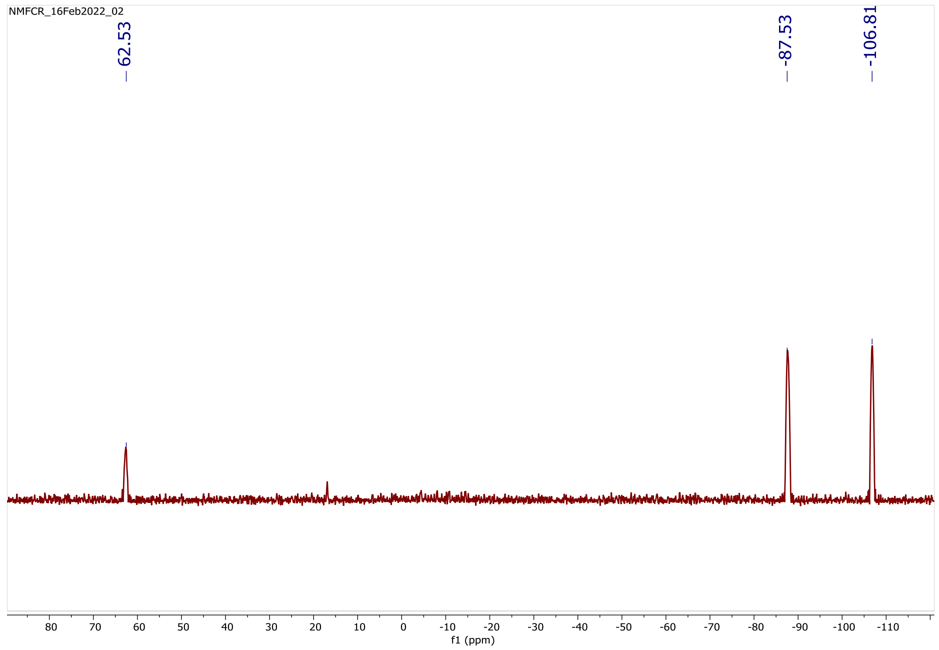

Figure S17 $^{11}B\{^{1}H\}$ NMR spectrum of [Na][CrSAN] in D_2O.

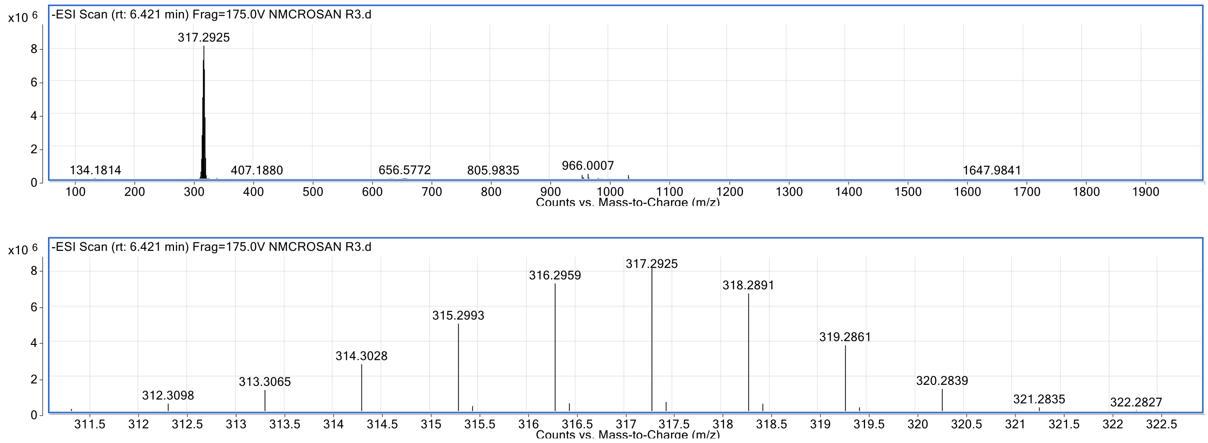

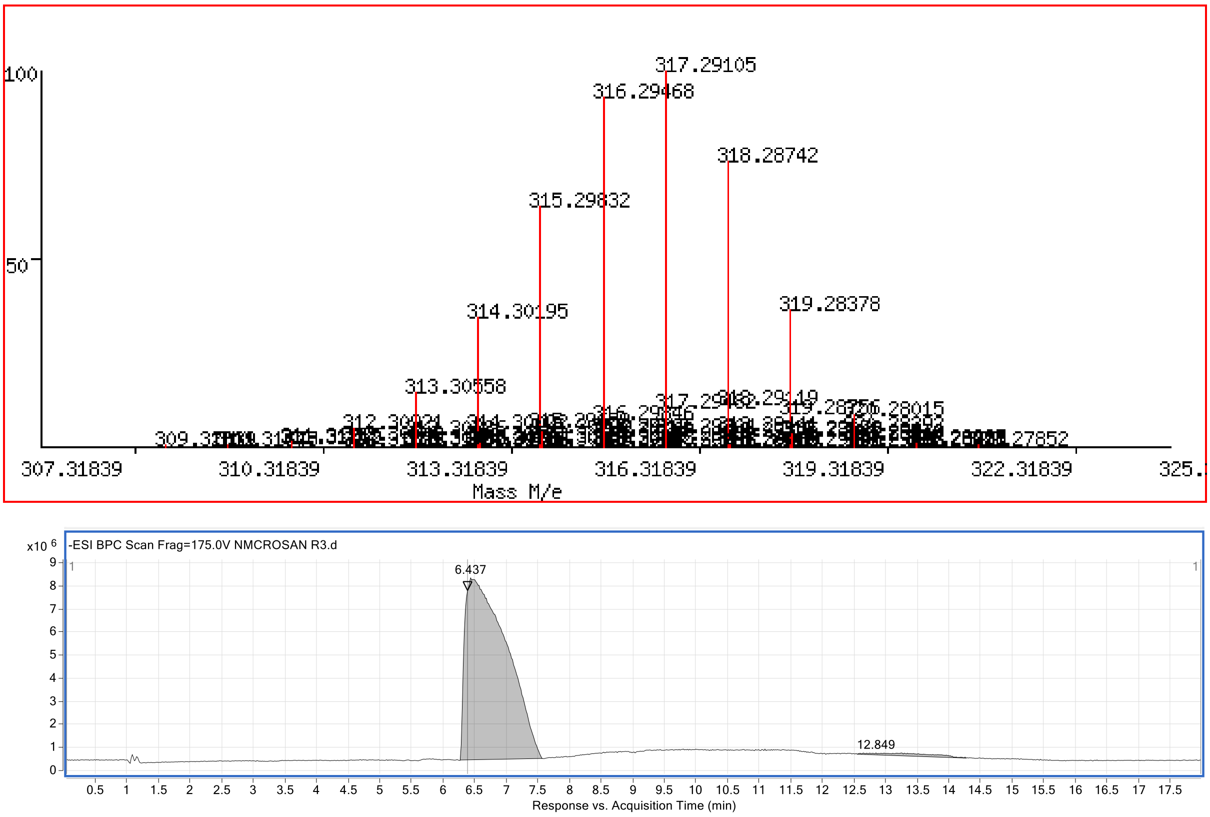

Figure S18 BPC scan and ESI peaks of [Na][CrSAN] (317.2925 m/z)

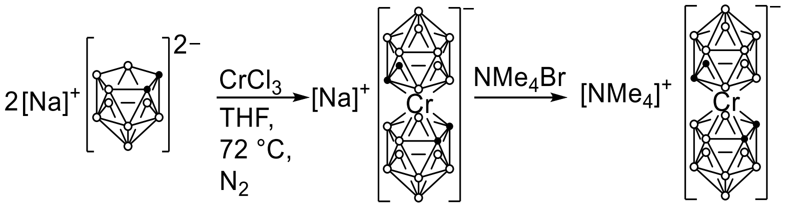

Scheme S2 Procedure for synthesis of Cr metallacarborane.

Preparation of partially deuterated cobalt (III) bis-dicarbollide [Na][CoSAN-D_2]

[Cs][CoSAN] (200 mg, 438 µmol) was dried *in vacuo* for four hours at 200 °C. Under nitrogen atmosphere, anhydrous DME (5 mL) was added and cooled to -40 °C using an acetonitrile bath cooled with liquid N_2. 2.5 M *n*-butyl lithium in hexanes (525 µL, 1.31 µmol) was then added dropwise to the solution with high stirring over 20 minutes. The mixture was allowed to warm slowly to room temperature for 30 minutes. Then, D_2O (1 mL) was added to the mixture via degassed syringe and stirred for a further 30 minutes. The solvent was filtered, removed *in vacuo* and the residue taken up in water. The sodium salt was prepared as described in the literature.[2] This was then extracted (x3 10 mL) with diethyl ether. The organic layer was retained, and the solvent removed under reduced pressure to give a dark orange residue. This was dried *in vacuo* to give [Na][CoSAN-D_2] (143.5 mg, 411.72 µmol, 94%). ^{1}H NMR (CD_3CN, 500 MHz): δ = 3.87 (3H, s), 3.67 (1H, s), 3.39 (1H, s), 3.06 (2H, s), 2.77 (2H, s), 2.49 (2H, s), 2.25 (2H, s), 2.14 (2H, s), 1.69 (3H, s), 1.41 (3H, s), 1.09 (1H, s) ^{11}B NMR (CD_3CN, 160.4 MHz): δ = 5.94 (s, 2B, 1J), 1.04 (s, 2B, 1J), -5.77 (d, 8B), -17.55 (s, 4B, 1J), -22.92 (s, 2B, 1J). ^{13}C NMR (CD_3CN, 125 MHz): δ 51.06 (s). FT-IR; 3599 cm^{-1} (s, b) 3042 cm^{-1} (w, b) (C-H) 2606

cm^{-1} (m) (B-H) 2572 cm^{-1} (s) (B-H) 2508 cm^{-1} (s) (B-H) 2271 cm^{-1} (w) (C-D) 1615 cm^{-1} (m,b) 1089 cm^{-1} (w) 1004 cm^{-1} (m) 889 (m). ESI-MS: *m/z* predicted: 326.28 found: 326.36.

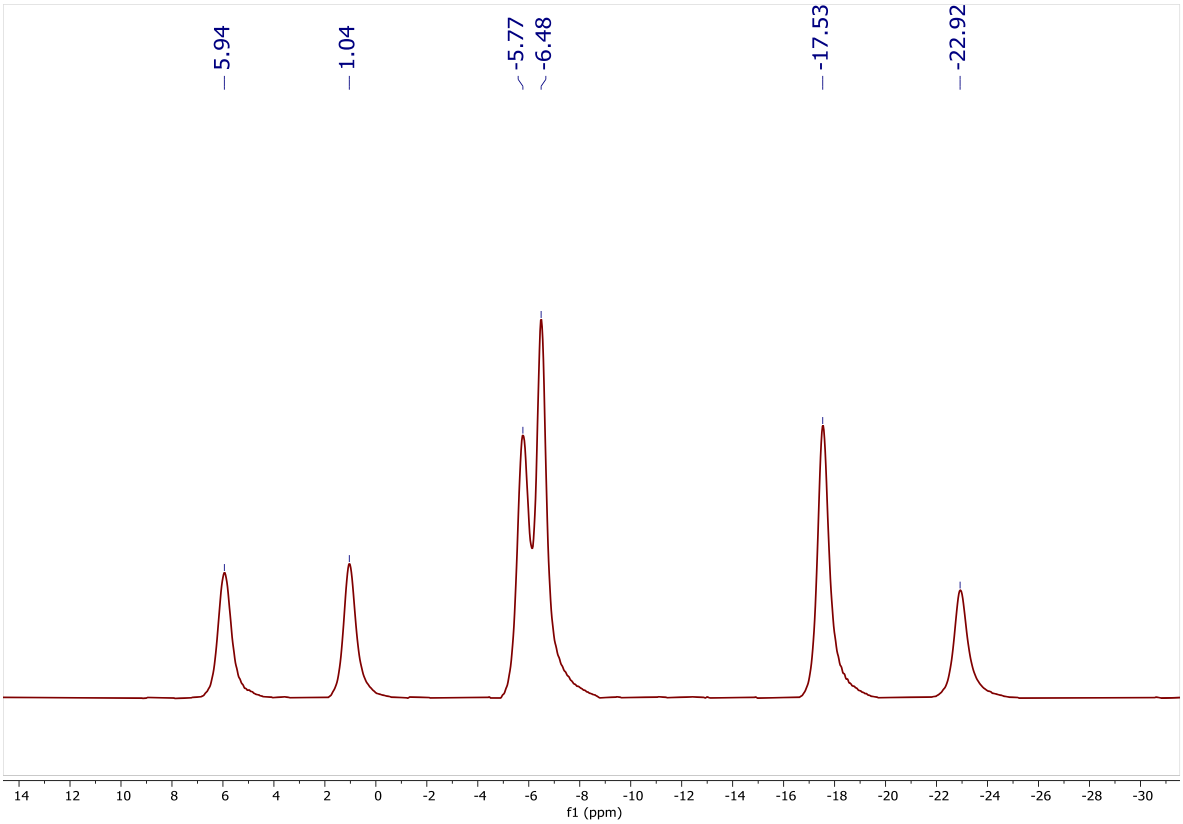

Figure S19 $^{11}B\{^{1}H\}$ NMR spectrum of [Na][CoSAN-D_2] in CD_3CN

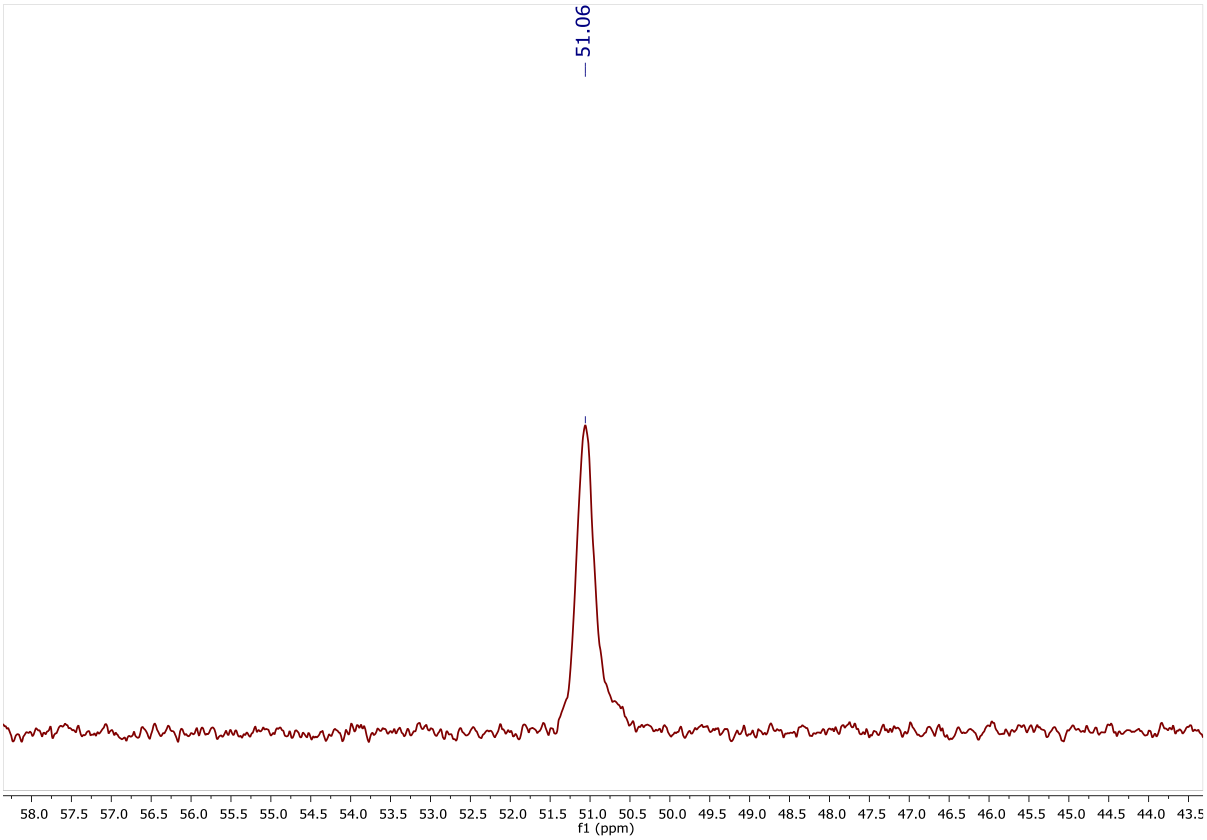

Figure S20 ^{13}C NMR spectrum of [Na][CoSAN-D_2] in CD_3CN

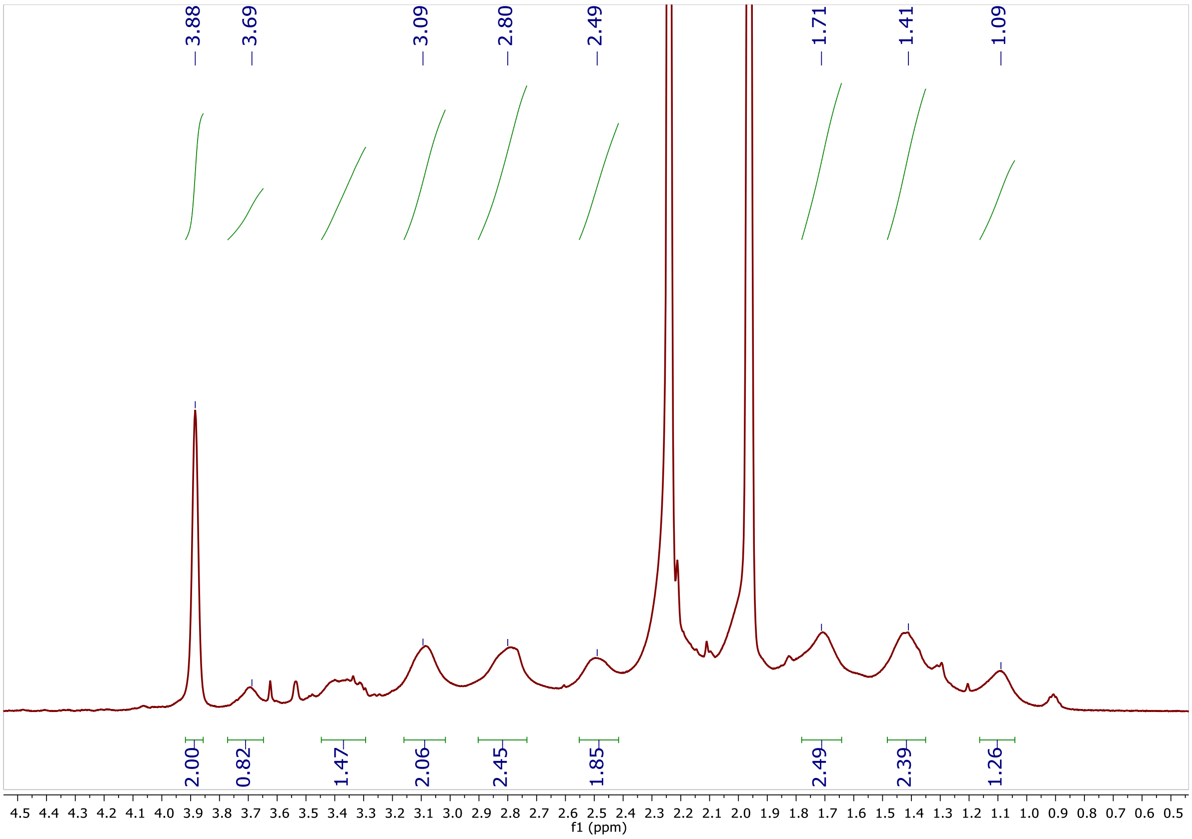

Figure S21 ^{1}H NMR spectrum of partially deuterated [Na][CoSAN-D_2] in CD_3CN.

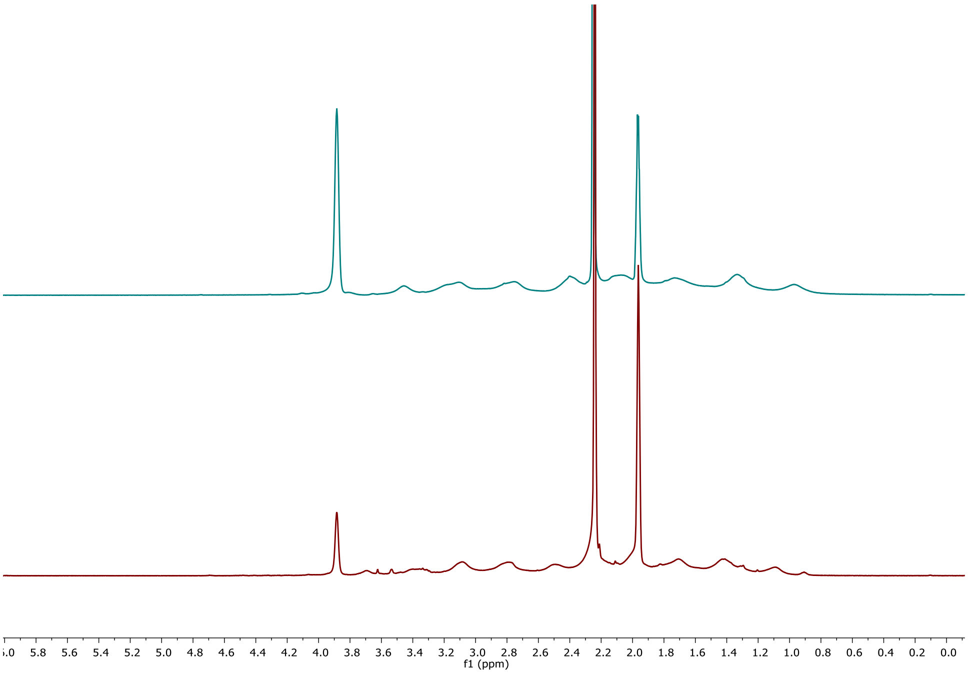

Figure S22 Stacked ^{1}H NMR spectra of [Na][CoSAN] (top) and partially deuterated [Na][CoSAN-D_2] (bottom) in CD_3CN.

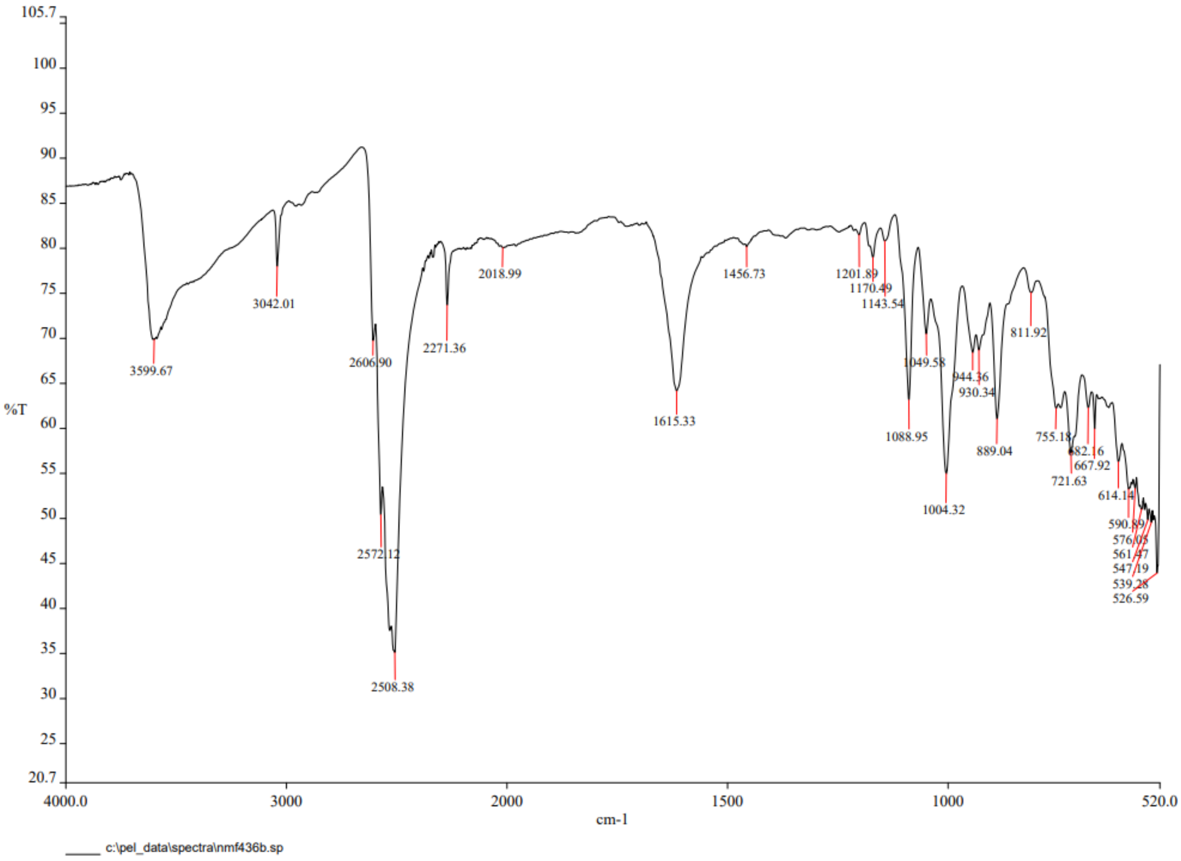

Figure S23 Infrared spectra of partially deuterated [Na][CoSAN-D_2]. B-H stretch at 2508 cm^{-1}, C-H stretch at 3042 cm^{-1} and C-D stretch at 2271 cm^{-1}.

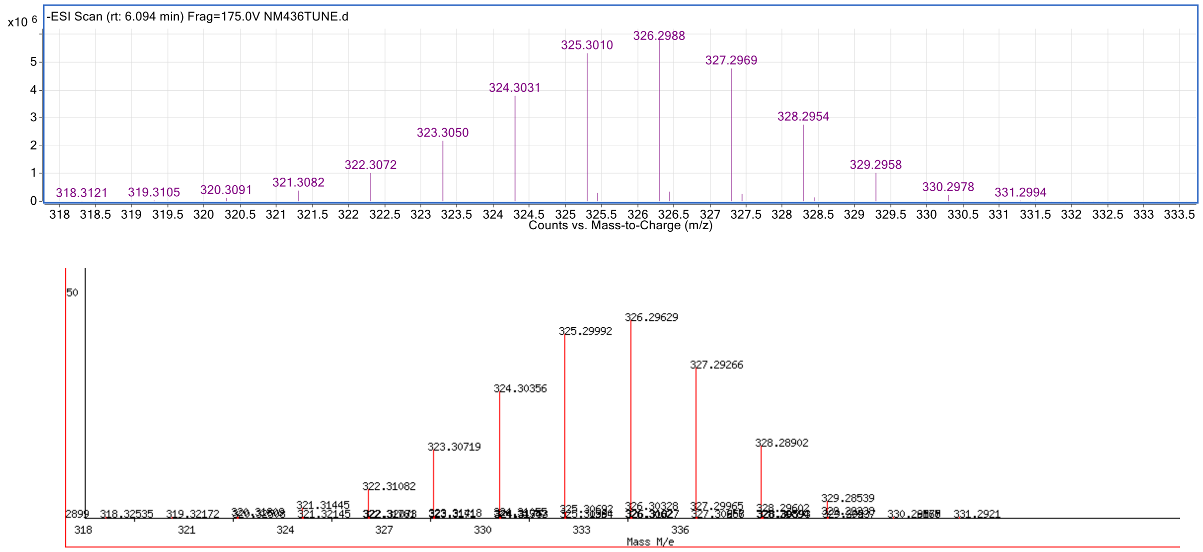

Figure S24 Mass spectrum of [Na][CoSAN-D_2] (top) with predicted distribution (bottom) (326.3566 m/z).

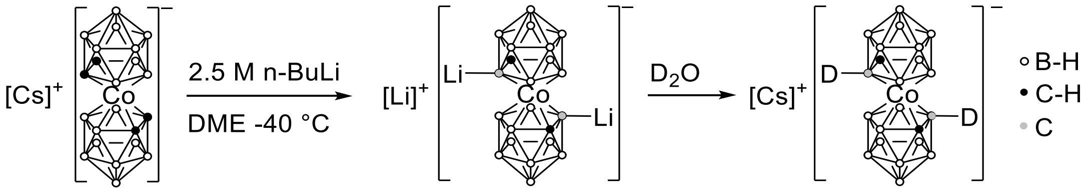

Scheme S3 Procedure for synthesis of deuterated cobalt (III) bis dicarbollide.

Isolation of sodium metallacarborane salts

Following a reported procedure Amberlite® IR-120 strongly acidic cation exchange resin was used to generate sodium salts of metallacarboranes.[2] Approximately 2/3 of a column was filled with the resin beads, and they were left in 3 M HCl overnight. It was then washed with 250 mL 3 M HCl and washed with deionised water until a neutral pH was obtained. Then, a 3 M NaCl solution is passed through the column until the eluent no longer has an acidic pH. The column is then washed with water until the Tollen's test indicates the column is free of salts. A solution of the desired tetramethylammonium metallacarborane salt is dissolved in 50:50 ACN:H_2O and then passed through the column three times. This is evaporated to dryness and extracted with brine/diethyl ether to remove salts. The organic solvent was removed under reduced pressure, the precipitate washed with cyclohexane and dried under vacuum.

References

1 M. F. Hawthorne, D. C. Young, T. D. Andrews, D. V. Howe, R. L. Pilling, A. D. Pitts, M. Reintjes, L. F. Warren and P. A. Wegner, *J. Am. Chem. Soc.*, 1968, **90**, 879–896.

2 I. Fuentes, A. Andrio, F. Teixidor, C. Vinas and V. Compana, *Phys. Chem. Chem. Phys.*, 2017, **4**, 15177–15186.

3 W. Ruhle, M. Frederick and M. Frederick, *Inorg. Chem.*, 1968, **7**, 9–12.
